# Temporal Regulation of UVA-Induced Ferroptosis-Associated Injury in Keratinocytes

**DOI:** 10.3390/ijms27208947

**Published:** 2026-10-09

**Authors:** Ren Dong, Xiang Li, Ping Wang, Qianxian Yin, Zirun Wang, Ying Xie

**Affiliations:** Key Laboratory of Molecular Epidemiology of Hunan Province, School of Public Health, Health Science Center, Hunan Normal University, Changsha 410013, China

**Keywords:** circadian rhythm, ferroptosis, glutathione peroxidase 4, HaCaT keratinocytes, oxidative stress, ultraviolet A

## Abstract

Ferroptosis contributes to ultraviolet (UV)-induced epidermal injury, but whether circadian state modifies keratinocyte susceptibility remains unclear. We integrated human epidermal transcriptomic analyses with functional experiments in HaCaT keratinocytes to compare UVA- and UVB-induced ferroptosis-associated responses and assess their temporal regulation. Both wavelengths reduced cell viability and increased reactive oxygen species, lipid peroxidation, and intracellular Fe^2+^ accumulation; ferrostatin-1 provided partial protection. UVB reduced xCT and GPX4 and showed a proteasome-sensitive component of GPX4 loss, whereas UVA predominantly reduced GPX4 without detectably altering xCT. Human epidermal transcriptomes showed heterogeneous cross-sectional associations among circadian, oxidative-stress, and ferroptosis-related genes, while longitudinal profiles revealed positive temporal coupling between *NR1D1*-associated signals and *GPX4*. In synchronized HaCaT cells, *ARNTL* and *NR1D1* displayed 24 h rhythmicity, whereas *GPX4* and *SLC7A11* did not meet the prespecified rhythmicity criterion. Nevertheless, UVA-induced cytotoxicity, oxidative stress, lipid peroxidation, and GPX4 loss varied across post-synchronization time points and were greatest at 24 h. SR8278 partially preserved GPX4 abundance and attenuated UVA-induced loss of viability, reactive oxygen species accumulation, and lipid peroxidation, without significantly affecting Fe^2+^ accumulation. These findings associate circadian state with temporal variation in UVA-induced ferroptosis susceptibility and implicate the broader clock-redox system rather than rhythmic expression of individual ferroptosis effectors.

## 1. Introduction

Circadian clocks coordinate metabolism, antioxidant defense, and stress responses across the 24 h cycle, while redox signals feedback on clock function [1]. Ferroptosis is an iron-dependent form of regulated cell death driven by membrane phospholipid peroxidation and controlled by iron, lipid, and antioxidant metabolism. Experimental studies in renal and airway epithelial models further suggest that clock state can modify ferroptosis susceptibility, although the responsible pathways are tissue and insult dependent [2,3].

Keratinocytes form the principal cellular barrier of the epidermis and contain an autonomous peripheral clock that contributes to epidermal homeostasis, proliferation, metabolism, and stress adaptation [4,5,6]. Exposed to solar ultraviolet (UV) radiation, high oxidative stress, and subject to intrinsic temporal gating, human keratinocytes serve as a key physiological model to address whether cellular timing shapes oxidative cell-injury trajectories and antioxidant defense stability.

The xCT–GSH–GPX4 axis functions as a primary ferroptosis-suppressive network. xCT (encoded by *SLC7A11*) imports cystine for glutathione (GSH) synthesis, enabling GPX4 to neutralize lipid hydroperoxides [7]. UVB can induce ferroptosis in keratinocytes and disturb glutathione-dependent antioxidant defense, thereby contributing to epidermal inflammation [8,9]. Notably, solar UVA and UVB exert distinct photochemical and biological impacts: UVB causes substantial direct DNA photodamage, whereas UVA penetrates more deeply and promotes photosensitized oxidant formation and lipid oxidation. Recent studies have linked UVA-induced damage to impaired NRF2/GPX4 signaling, iron overload, and lipid peroxidation [10]. Given the central roles of cystine availability and GPX4-mediated lipid peroxide detoxification in maintaining this ferroptosis-suppressive system, comparing UVA and UVB provides a rationale for determining whether these distinct wavelengths affect the xCT–GSH–GPX4 axis differently. However, whether UVA and UVB produce distinct or shared alterations in this axis remains unclear.

Furthermore, it remains unknown whether a fixed UVA challenge elicits different ferroptosis-associated responses at distinct post-synchronization states, and whether circadian state is associated with changes in GPX4-centered redox defense in keratinocytes. Cutaneous circadian clock function can also be perturbed by, or interact with, UV exposure. Low-dose UVA1 alters CRY2 protein and clock-related transcripts in human skin [11], while the circadian amplitude enhancer nobiletin displays differential cell-survival effects following UVA versus UVB irradiation [12]. These observations suggest that keratinocyte responses to different UV wavelengths may vary according to cellular temporal state. HaCaT keratinocytes have been established as a robust in vitro model for studying cell-autonomous circadian clock function, with experimentally synchronized rhythms of canonical clock genes [13]. We hypothesized that the post-synchronization temporal state of HaCaT cells modifies their susceptibility to UVA-induced ferroptosis-associated injury and that this temporal variation is accompanied by changes in GPX4-centered redox defense.

To test this hypothesis, we systematically compared the distinct impacts of UVA and UVB exposure on the xCT/GPX4 defense system in human keratinocytes and evaluated whether UVA-induced defense impairment exhibits time-dependent variations following clock synchronization. By analyzing longitudinal human skin transcriptomes, we sought to nominate circadian-associated candidate genes within the cutaneous clock network. Finally, using the REV-ERB ligand SR8278 as a pharmacological probe, we explored whether targeting circadian clock components can attenuate UVA-induced GPX4 depletion and lipid peroxidation, interpreting these responses in light of both REV-ERB-dependent and non-target-mediated pharmacological actions.

## 2. Results

### 2.1. UVA and UVB Induce Ferroptosis-Associated Injury in HaCaT Keratinocytes

**Figure 1:**
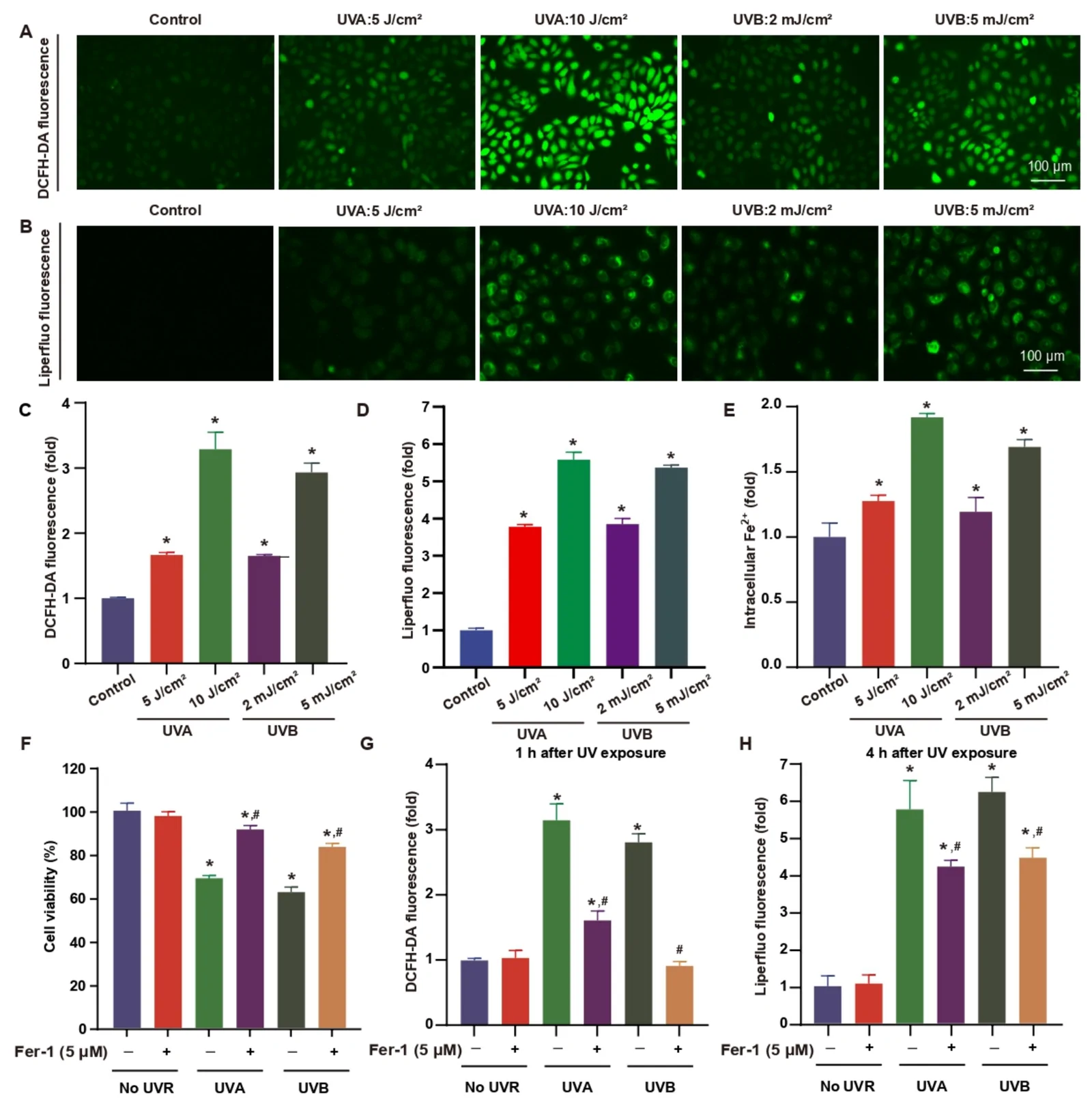
UVA and UVB induce ferroptosis-associated injury in HaCaT keratinocytes. (**A**–**D**) Representative images and quantification of DCFH-DA fluorescence at 1 h (**A**,**C**) and Liperfluo fluorescence at 4 h (**B**,**D**) following the indicated doses of UVA or UVB irradiation. Scale bars, 100 μm. (**E**) Intracellular labile Fe^2+^ levels measured at 2 h after irradiation. For (**C**–**E**), statistical significance was assessed by ordinary one-way ANOVA followed by Šídák-adjusted prespecified comparisons. (**F**–**H**) Cell viability at 48 h (**F**), DCFH-DA fluorescence at 1 h (**G**), and Liperfluo fluorescence at 4 h (**H**) following UVA (10 J/cm^2^) or UVB (5 mJ/cm^2^) irradiation in the presence or absence of Fer-1 (5 μM). Values were normalized to the corresponding non-irradiated controls. For (**F**–**H**), ordinary two-way ANOVA with UV condition and Fer-1 treatment as factors was used, followed by Šídák-adjusted prespecified comparisons. Data are presented as mean ± SD from three independent experiments (*n* = 3). * *p* < 0.05 versus the corresponding non-irradiated control; # *p* < 0.05 versus the corresponding UV-only group.

To characterize the cellular injury induced by different ultraviolet (UV) spectra, HaCaT keratinocytes were exposed to graded doses of UVA or UVB irradiation. Both UVA and UVB reduced cell viability in a dose- and time-dependent manner (Figure S1A,B). Intracellular reactive oxygen species (ROS) accumulation was rapidly increased within 1 h after irradiation (Figure 1A,C), followed by a marked increase in lipid peroxidation at 4 h (Figure 1B,D). In parallel, intracellular labile Fe^2+^ levels were significantly elevated at 2 h after irradiation (Figure 1E), suggesting the involvement of iron-dependent oxidative stress in UV-induced cellular injury.

To determine whether ferroptosis-associated mechanisms contribute to UV-induced cytotoxicity, cells were treated with ferrostatin-1 (Fer-1), a lipophilic ferroptosis inhibitor, in combination with UVA or UVB irradiation. Fer-1 at a non-cytotoxic concentration (up to 5 μM; Figure S1C) significantly restored cell viability and attenuated ROS accumulation and lipid peroxidation following both UVA and UVB exposure (Figure 1F–H). Collectively, these findings indicate that ferroptosis-associated oxidative injury contributes to UVA- and UVB-induced damage in HaCaT keratinocytes.

### 2.2. Differential Regulation of the xCT–GPX4 Axis by UVA and UVB

**Figure 2:**
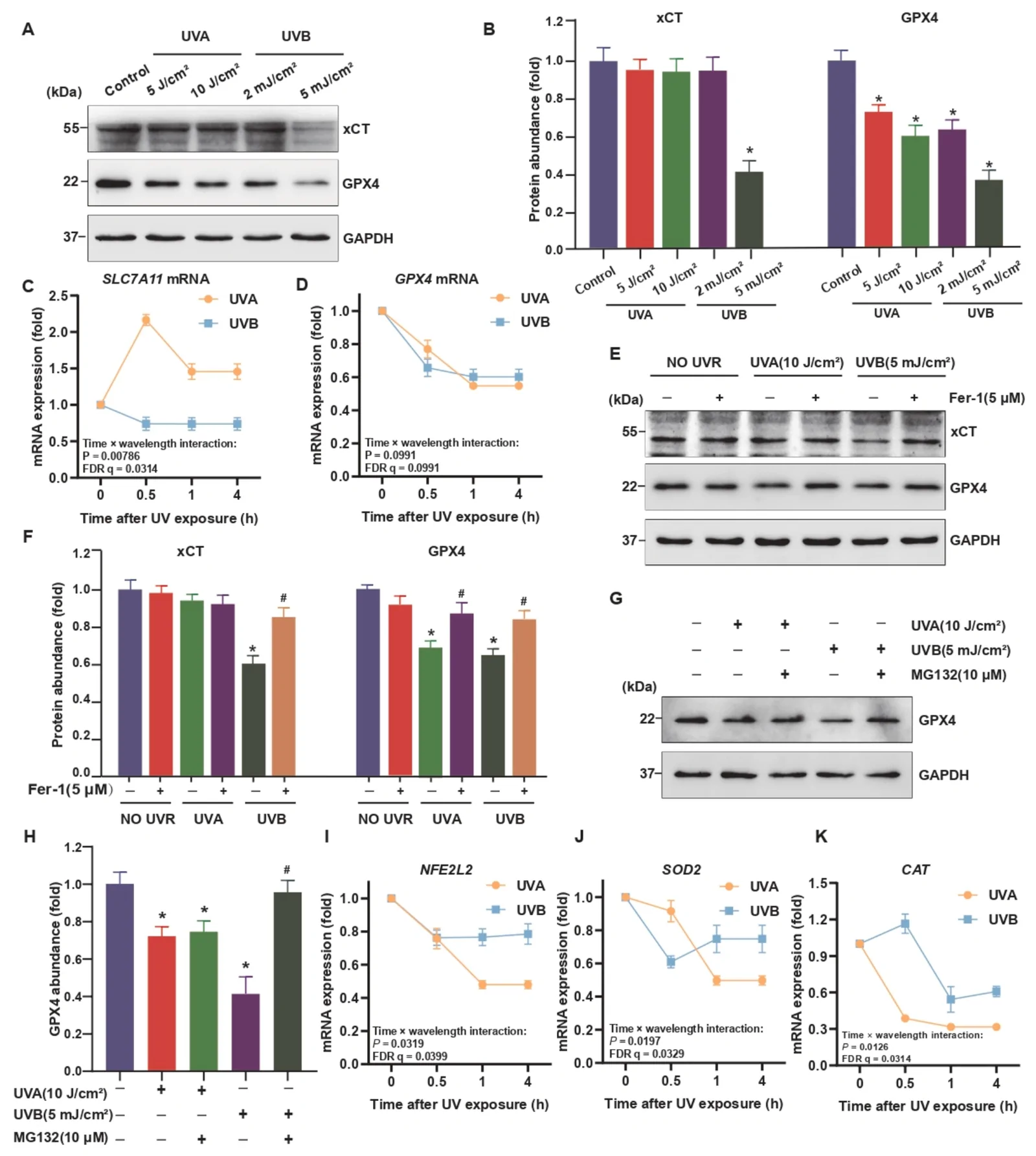
UVA and UVB differentially regulate the xCT–GPX4 antioxidant-defense axis in HaCaT keratinocytes. (**A**,**B**) xCT and GPX4 protein abundance following graded UVA or UVB irradiation. (**C**,**D**) *SLC7A11* and *GPX4* mRNA expression at 0–4 h after UVA or UVB irradiation. (**E**,**F**) xCT and GPX4 protein abundance following UV irradiation in the presence or absence of Fer-1. (**G**,**H**) GPX4 protein abundance following UV irradiation in the presence or absence of MG132. (**I**–**K**) *NFE2L2*, *SOD2*, and *CAT* mRNA expression at 0–4 h after irradiation. For protein analyses, samples were collected 24 h after irradiation. For (**B**,**F**,**H**), statistical significance was assessed by ordinary one-way ANOVA followed by Šídák-adjusted prespecified comparisons. For (**C**,**D**,**I**–**K**), models were fitted to raw −ΔCt values with wavelength, time, and their interaction as fixed effects and batch as the repeated-measures subject; expression levels are displayed as 2^−ΔΔCt^. *p* values indicate time × wavelength interactions. FDR q values were calculated across the five prespecified genes using the Benjamini–Hochberg procedure. Data are presented as mean ± SD from three independent experiments (*n* = 3). * *p* < 0.05 versus the corresponding non-irradiated control; # *p* < 0.05 versus the corresponding UV-only group.

Given the central role of the xCT–GPX4 axis in ferroptosis defense, we examined its response to UVA and UVB irradiation at both the protein and transcript levels. Both UVA and UVB reduced GPX4 protein abundance, whereas xCT protein was markedly decreased by UVB but minimally affected by UVA (Figure 2A,B). Consistent with these findings, *SLC7A11* mRNA was not significantly reduced by UVA. Nevertheless, *SLC7A11* showed a significant time × wavelength interaction (q = 0.0314), indicating wavelength-dependent temporal responses. In contrast, *GPX4* mRNA was suppressed by both UV spectra without a significant time × wavelength interaction (Figure 2C,D). These results indicate distinct effects of UVA and UVB on the xCT–GPX4 axis.

We next examined whether altered protein stability contributed to the differential regulation of xCT and GPX4. Fer-1 partially preserved GPX4 protein levels following UVA irradiation and restored both xCT and GPX4 levels following UVB exposure (Figure 2E,F). Moreover, the proteasome inhibitor MG132 substantially attenuated UVB-induced GPX4 loss but had little effect following UVA exposure (Figure 2G,H), suggesting that a proteasome-sensitive component is involved in the UVB response. However, this pharmacological experiment does not identify the specific pathway responsible for GPX4 loss.

We further assessed key antioxidant response genes and identified significant time × wavelength interactions for *NFE2L2*, *SOD2*, and *CAT*, with Benjamini–Hochberg-adjusted q values of 0.0399, 0.0329, and 0.0314, respectively (Figure 2I–K). UVA produced a more pronounced reduction in *NFE2L2* and *CAT* expression than UVB. Collectively, these findings demonstrate that UVA and UVB differentially perturb the xCT–GPX4 antioxidant defense system, with distinct effects on antioxidant transcriptional responses and protein-level regulation.

### 2.3. Human Skin Transcriptomics Reveals Dynamic Temporal Coupling Between GPX4 and Circadian Genes

**Figure 3:**
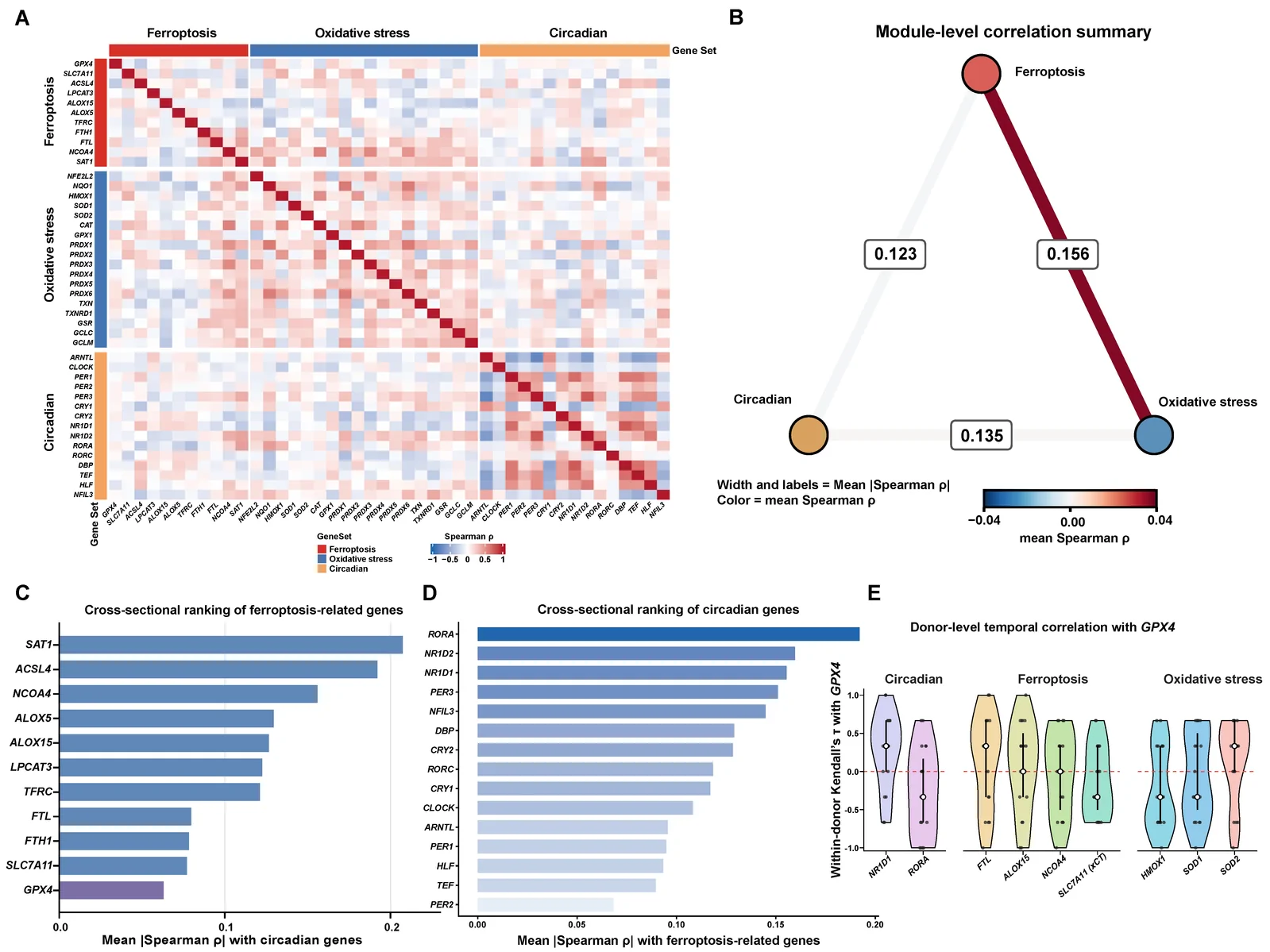
Human epidermal transcriptomes reveal context-dependent associations between circadian and ferroptosis-related genes. (**A**) Spearman correlation matrix of 11 ferroptosis-related, 18 oxidative-stress, and 15 circadian genes across 219 independent cross-sectional forearm epidermis samples from GSE112660. Of 946 pairwise gene correlations, 420 remained significant after Benjamini–Hochberg FDR correction (FDR < 0.05). White cells indicate non-significant associations. (**B**) Summary of between-module correlations. Edge color represents mean signed Spearman ρ, whereas edge width and labels indicate mean |ρ|. (**C**) Ferroptosis-related genes ranked by their mean |ρ| with the circadian gene set. (**D**) Circadian genes ranked by their mean |ρ| with the ferroptosis-related gene set. (**E**) Within-donor Kendall’s τ values between *GPX4* and representative genes across four longitudinal epidermal samples from 19 donors with complete profiles. Each point represents one donor; white circles indicate medians, black vertical lines indicate interquartile ranges, and violin plots show the distributions. Donor-level Kendall’s τ distributions were tested against zero using exact two-sided Wilcoxon signed-rank tests, with Benjamini–Hochberg correction across the nine prespecified candidate genes.

In 219 independent cross-sectional forearm epidermis samples, correlations among the prespecified ferroptosis-related, oxidative-stress, and circadian genes were heterogeneous (Figure 3A). Module-level correlation analysis revealed detectable associations among the three biological programs. The mean absolute correlation was 0.156 between ferroptosis and oxidative stress, 0.123 between ferroptosis and circadian rhythm, and 0.135 between oxidative stress and circadian rhythm (Figure 3B). These cross-sectional, unadjusted correlations describe gene expression associations at the population level and do not establish temporal ordering or causal regulation.

Next, we examined the contribution of individual genes to the associations between these functional modules. Among the 11 selected ferroptosis-related genes, *SAT1* and *ACSL4* showed the highest mean |ρ| values with the circadian gene set (0.207 and 0.192, respectively), whereas *GPX4* showed the lowest mean |ρ| value (0.063; Figure 3C). Conversely, among the selected circadian genes, *RORA*, *NR1D2*, and *NR1D1* showed the highest mean |ρ| values in relation to the ferroptosis-related gene set (0.192, 0.160, and 0.155, respectively; Figure 3D). To further examine the temporal relationships between these functional modules, we analyzed an independent donor-level longitudinal dataset to assess gene expression changes across different time points within the same donor. Representative genes from the three functional modules were selected, and their within-donor temporal associations with *GPX4* were evaluated (Figure 3E). Among the circadian genes, *NR1D1* showed a positive median Kendall’s τ with *GPX4*, whereas *RORA* showed a negative median Kendall’s τ, indicating opposite directions of temporal co-variation with *GPX4*. Other selected genes showed heterogeneous temporal associations with *GPX4* (Figure 3E).

### 2.4. GPX4 Lacks a Detectable Basal 24 h Rhythm in Synchronized HaCaT Cells

**Figure 4:**
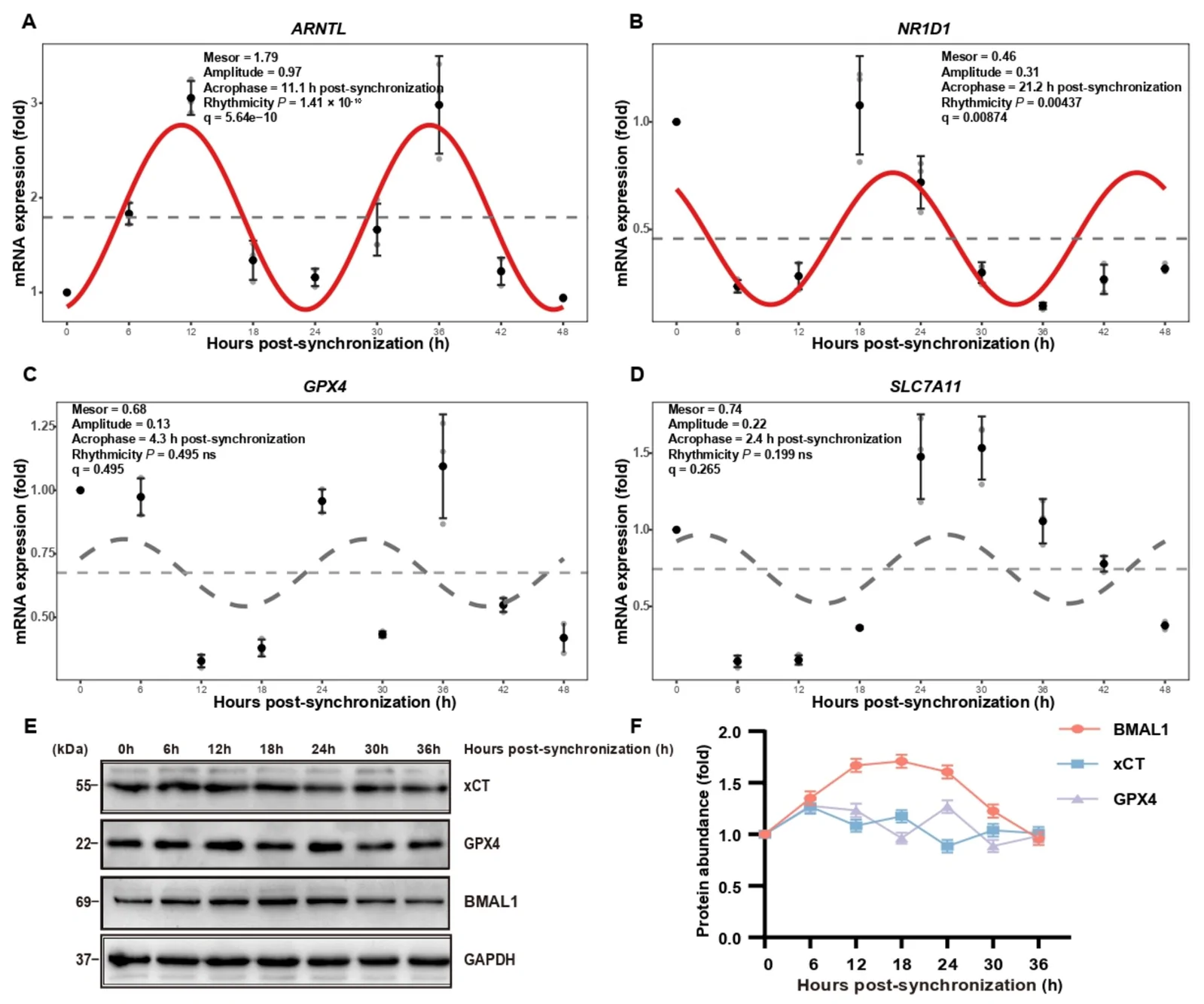
GPX4 and xCT lack detectable basal 24 h rhythmicity in synchronized HaCaT cells. (**A**–**D**) *ARNTL*, *NR1D1*, *GPX4*, and *SLC7A11* mRNA expression following dexamethasone synchronization, with fixed 24 h cosinor fits. Solid curves indicate statistically significant 24 h cosinor fits, whereas gray dashed curves represent nonsignificant cosinor fits. (**E**,**F**) BMAL1, GPX4, and xCT protein abundance over 0–36 h. Rhythmicity *p* values were obtained from joint tests of the fixed 24 h cosine and sine terms. FDR-adjusted q values were calculated across the four prespecified genes using the Benjamini–Hochberg procedure.

Dexamethasone synchronization induced robust 24 h rhythmic expression of the canonical clock genes *ARNTL* and *NR1D1*, whereas *GPX4* and *SLC7A11* mRNA did not meet the prespecified criteria for circadian rhythmicity (Figure 4A–D). At the protein level, BMAL1 abundance varied over the 0–36 h time course and showed a temporal pattern broadly consistent with its mRNA expression, whereas neither GPX4 nor xCT exhibited a stable 24 h oscillatory pattern (Figure 4E,F). Thus, under the conditions tested, GPX4 did not exhibit a detectable basal 24 h oscillation in synchronized HaCaT cells.

### 2.5. UVA-Induced Injury in HaCaT Keratinocytes Varies Across the Post-Synchronization Time Course

**Figure 5:**
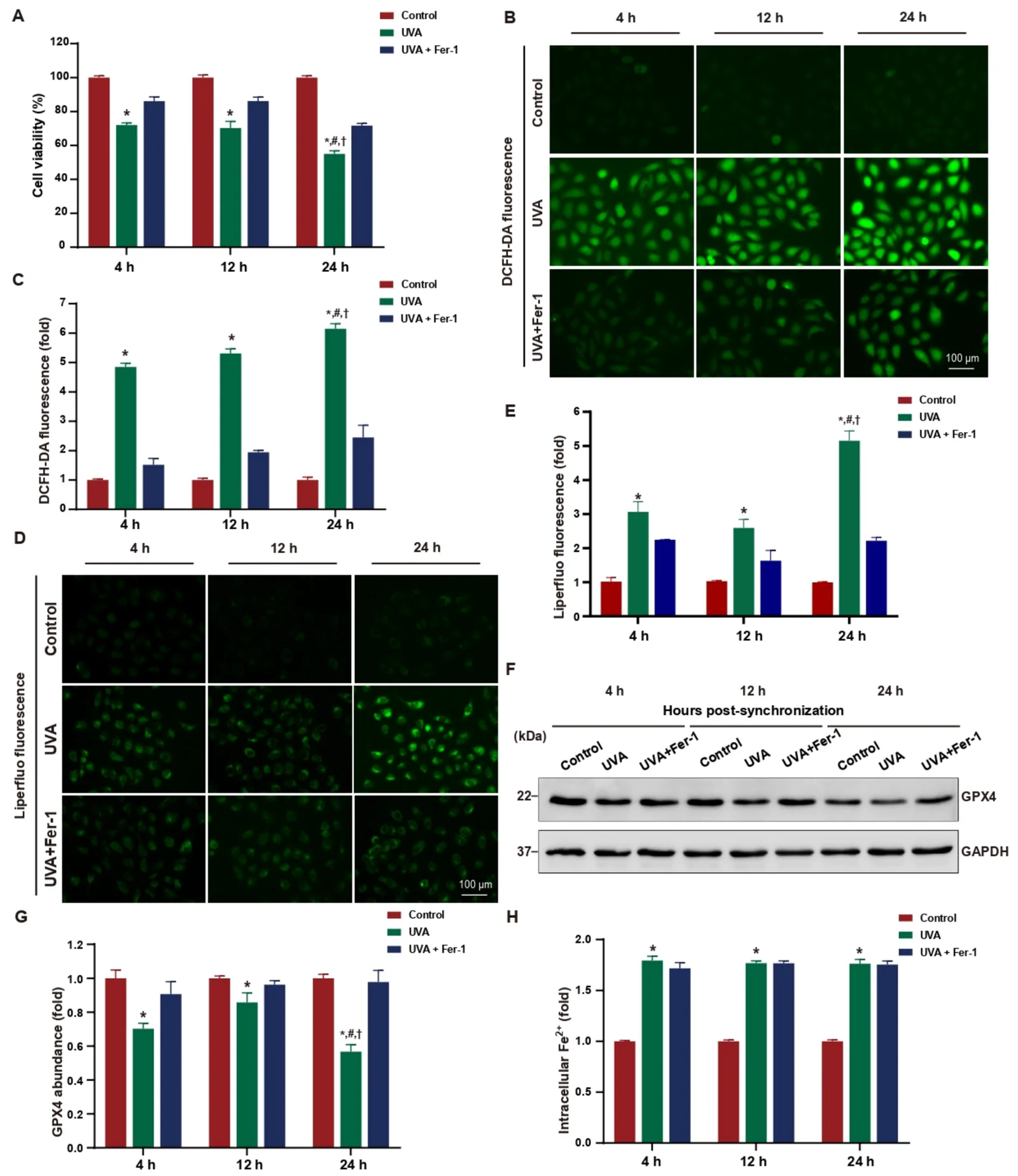
UVA-induced injury in HaCaT keratinocytes varies across the post-synchronization time course. Synchronized cells were exposed to UVA (10 J/cm^2^) at 4, 12, or 24 h post-synchronization. (**A**) Cell viability at 48 h. (**B**,**C**) DCFH-DA fluorescence at 1 h. (**D**,**E**) Liperfluo fluorescence at 4 h. (**F**,**G**) GPX4 protein abundance at 24 h. (**H**) Intracellular Fe^2+^ levels at 2 h. Fer-1 (5 μM) was included where indicated. Values were normalized to the matched non-irradiated control and are presented as mean ± SD from three independent biological replicates (*n* = 3). Statistical significance was assessed by ordinary two-way ANOVA with time and treatment as factors. *p* values for the time × treatment interaction were adjusted across five prespecified endpoints using the Benjamini–Hochberg procedure. Šídák-adjusted multiple comparisons were used for prespecified pairwise comparisons between UVA and the matched control group at each time point and among time points within the UVA group. * *p* < 0.05 versus the matched control group at the same post-synchronization time; ^#^ *p* < 0.05 for 24 h UVA versus 4 h UVA; ^†^ *p* < 0.05 for 24 h UVA versus 12 h UVA.

To determine whether the timing of UVA exposure after synchronization affects keratinocyte susceptibility, synchronized HaCaT cells were exposed to a fixed dose of UVA at 4, 12, or 24 h post-synchronization, and cellular responses were assessed at the prespecified assay time points. Despite the absence of a detectable basal 24 h rhythm in GPX4, UVA responses varied across the post-synchronization time course. UVA significantly reduced cell viability and GPX4 abundance and increased ROS accumulation and lipid peroxidation compared with matched control groups at 4, 12, and 24 h (Figure 5A–G). Within the UVA group, cell viability and GPX4 abundance were significantly lower, whereas DCFH-DA and Liperfluo fluorescence were significantly higher, at 24 h than at 4 and 12 h. Fer-1 partially attenuated the UVA-induced changes in cell viability, ROS, and lipid peroxidation, supporting the involvement of ferroptosis-related oxidative injury. In contrast, UVA significantly increased intracellular Fe^2+^ levels at all three time points, with no significant differences among 4, 12, and 24 h within the UVA group (Figure 5H). Together, these findings indicate that UVA-induced changes in cell viability, oxidative stress, lipid peroxidation, and GPX4 abundance were most pronounced at 24 h after synchronization.

### 2.6. UVA Selectively Reconfigures Temporal Clock and Redox Transcriptional Programs in Synchronized HaCaT Cells

**Figure 6:**
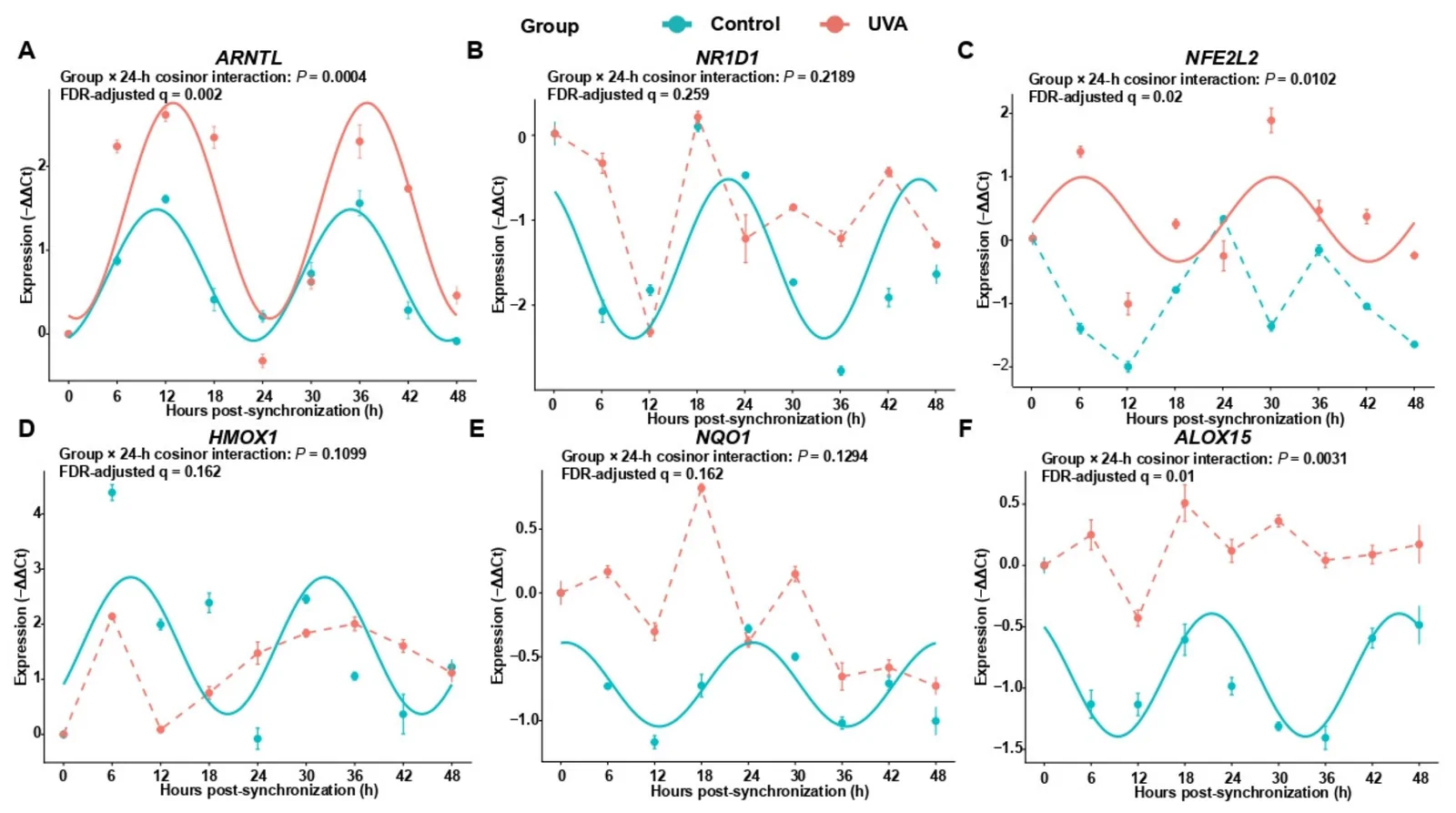
UVA selectively reconfigures temporal clock and redox expression patterns. Synchronized HaCaT cells were irradiated with UVA (10 J/cm^2^) at 0 h and sampled every 6 h through 48 h. Panels show *ARNTL* (**A**), *NR1D1* (**B**), *NFE2L2* (**C**), *HMOX1* (**D**), *NQO1* (**E**), and *ALOX15* (**F**). Data are presented as mean ± SE from three independent batches (*n* = 3), and values are displayed as −ΔΔCt. Models were fitted to −ΔCt. Solid curves indicate significant 24 h cosinor fits; dashed lines indicate no significant 24 h rhythmicity detected, with no fitted cosinor curve displayed. *p* values indicate group × cosinor interaction effects, and FDR-adjusted q values were calculated across the 10 prespecified genes in Figure 6 and Figure S2 using the Benjamini–Hochberg procedure.

In synchronized HaCaT cells, UVA exposure was associated with gene-specific alterations in the temporal expression patterns of circadian clock and redox-related genes rather than a generalized loss of rhythmicity. *ARNTL* retained significant 24 h rhythmicity in both control and UVA-exposed cells, while its fitted temporal pattern differed significantly between groups (Figure 6A). Similarly, *CLOCK* and *PER2* remained rhythmic in both groups but exhibited significant differences in their temporal patterns between conditions. In contrast, *CRY2* remained rhythmic in both groups, whereas *RORA* met the rhythmicity criterion in control cells but not following UVA exposure (Figure S2). Notably, *NR1D1* met the prespecified rhythmicity criterion in controls but not following UVA exposure, although its group × cosinor interaction was not significant (Figure 6B). Its circadian role and positive temporal association with *GPX4* in human epidermis motivated its selection for subsequent pharmacological evaluation.

Redox- and ferroptosis-related genes likewise showed gene-specific temporal responses to UVA. *NFE2L2* displayed rhythmicity specifically following UVA exposure, whereas *HMOX1*, *NQO1*, and *ALOX15* were rhythmic only in control cells (Figure 6C–F). Among these genes, *NFE2L2* and *ALOX15* also showed evidence of altered rhythmic patterns between control and UVA-exposed cells, whereas no such difference was detected for *HMOX1* or *NQO1*. Detailed group-specific cosinor parameters, including mesor, amplitude, acrophase, and FDR-adjusted rhythmicity and group × cosinor interaction *p* values, are provided in Supplementary Table S3. Together, these findings indicate that UVA selectively reconfigures the temporal organization of circadian and redox-related transcriptional programs, rather than uniformly disrupting their rhythmicity.

### 2.7. SR8278 Partially Attenuates UVA-Associated Oxidative and Lipid-Peroxidative Responses in HaCaT Cells

**Figure 7:**
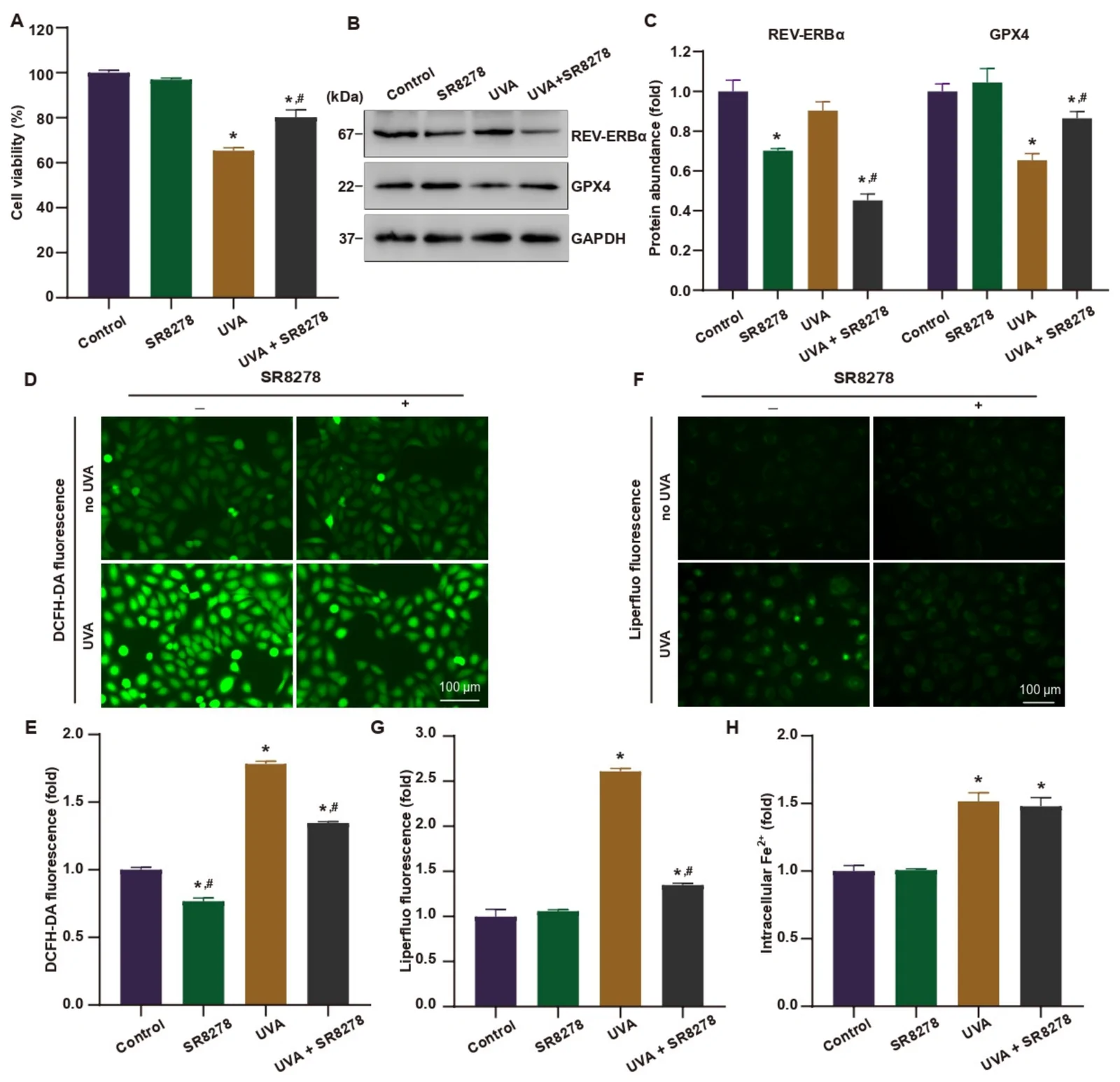
SR8278 partially attenuates UVA-associated oxidative and lipid-peroxidative injury. Synchronized HaCaT cells were pretreated with SR8278 (10 μM, 12 h) before UVA irradiation (10 J/cm^2^). (**A**) Cell viability at 48 h. (**B**,**C**) REV-ERBα and GPX4 protein abundance at 24 h. (**D**,**E**) DCFH-DA fluorescence at 1 h. (**F**,**G**) Liperfluo fluorescence at 4 h. (**H**) Intracellular Fe^2+^ levels at 2 h. Values were normalized to the non-irradiated control and are presented as mean ± SD from three independent experiments (*n* = 3). Statistical significance was assessed by ordinary one-way ANOVA followed by Šídák-adjusted prespecified comparisons. * *p* < 0.05 versus control; # *p* < 0.05 versus UVA alone.

The positive within-donor temporal association between *NR1D1* expression and *GPX4* in human epidermis, together with the observation that *NR1D1* met the rhythmicity criterion in synchronized HaCaT cells under control conditions but not following UVA exposure, prompted exploratory pharmacological evaluation of REV-ERBα-associated signaling using SR8278. We therefore used SR8278, a pharmacological inhibitor reported to interfere with REV-ERBα activity [14], to assess whether pharmacological perturbation of this pathway altered the GPX4-related response. SR8278 alone did not significantly affect cell viability, whereas pretreatment partially attenuated UVA-induced viability loss (Figure 7A). SR8278 reduced basal REV-ERBα abundance, which was further reduced following combined UVA and SR8278 treatment. Notably, although UVA decreased GPX4 abundance, SR8278 partially preserved GPX4 protein levels following UVA exposure (Figure 7B,C).

SR8278 also reduced basal DCFH-DA fluorescence and attenuated both DCFH-DA and Liperfluo fluorescence following UVA exposure, without significantly affecting intracellular Fe^2+^ accumulation (Figure 7D–H). These findings support a role for REV-ERBα-associated signaling in the regulation of GPX4 protein abundance during UVA stress, with its pharmacological modulation accompanied by reduced lipid peroxidation and cellular injury, but not by a measurable change in Fe^2+^ accumulation.

## 3. Discussion

This study identifies temporal variation in the susceptibility of keratinocytes to UVA-associated ferroptosis-related injury and provides evidence that this variation cannot be explained simply by rhythmic expression of a single ferroptosis effector. The initial comparison of UVA and UVB established that both wavelengths elicited a ferroptosis-associated oxidative injury phenotype, while also revealing distinct alterations in the xCT–GPX4 antioxidant defense system. This distinction was particularly evident for UVA, which was associated with GPX4 loss without a corresponding reduction in xCT protein, suggesting that UVA and UVB engage partially different routes of impairment within the same redox defense axis. Importantly, basal GPX4 abundance itself did not show a reproducible 24 h rhythmic pattern in synchronized keratinocytes. Nevertheless, the cellular response to a fixed UVA dose varied substantially across post-synchronization time points, with corresponding changes in GPX4 loss and lipid peroxidation. These findings shift the focus from asking whether an individual ferroptosis-related protein oscillates over the day to considering whether the broader cellular state established by the clock–redox system influences the magnitude of UVA-associated injury. The partial protection afforded by REV-ERBα modulation further supports this interpretation and provides a potential molecular entry point for understanding how clock-associated signaling may shape redox defense during UVA stress.

The comparison between UVA and UVB suggests that ferroptosis-associated injury is not mediated through an identical redox response to different wavelengths. The UVB phenotype observed here is consistent with previous evidence that UVB can impair the xCT–GSH–GPX4 antioxidant defense system and contribute to ferroptosis-related keratinocyte injury and cutaneous inflammation [8,9]. In the present study, UVB reduced both xCT and GPX4, whereas UVA produced a different pattern characterized by GPX4 loss without a detectable reduction in xCT protein and with a transient increase in *SLC7A11* mRNA. Thus, although both wavelengths converged on oxidative and lipid-peroxidative injury, the accompanying changes in the xCT–GPX4 axis were wavelength-dependent. The response to MG132 provided further evidence for this distinction. Proteasome inhibition attenuated GPX4 loss after UVB but had little effect after UVA, consistent with the involvement of proteasome-sensitive protein turnover in the UVB response, while suggesting that GPX4 loss following UVA involves additional or alternative mechanisms. Because GPX4 abundance can be influenced by multiple post-translational and protein-turnover pathways [15,16,17], these findings do not identify the precise mechanism responsible for UVA-associated GPX4 loss. Rather, they indicate that the UVA response cannot be fully understood by extrapolating mechanisms established for UVB and warrant further investigation of how UVA perturbs GPX4 protein homeostasis.

Given the distinct mechanistic signature of UVA injury, it is necessary to consider its cellular impact within a diurnal framework. The skin harbors a functional peripheral circadian clock that contributes to the local coordination of epidermal homeostasis, barrier function, and cellular responses to environmental stress [5,6]. Indeed, primary evidence demonstrates a time-of-day-dependent sensitivity (temporal gating) to UV radiation, where acute solar UV-B exposure leads to distinct diurnal variations in sunburn erythema and cutaneous injury [18]. This temporal organization is particularly relevant to photo-induced stress, as solar radiation represents a recurrent environmental exposure that interacts with the molecular clock over the course of daily life. Consistent with this concept, chronically photoexposed human skin in vivo exhibits fewer rhythmic transcripts, reduced oscillation amplitudes, and phase advances compared with photoprotected skin, indicating that persistent photoexposure is associated with remodeling of the temporal organization of epidermal gene expression [19]. Together, these observations provide a biological basis for asking whether ferroptosis-related redox responses to UVA also vary according to the temporal state of keratinocytes. We therefore considered the relationship between circadian, oxidative-stress, and ferroptosis-related programs at the level of human epidermal gene expression before examining temporal UVA responses experimentally.

The human epidermal transcriptomes provided an essential baseline framework for understanding how circadian, oxidative stress, and ferroptotic pathways intersect in cutaneous tissue. Across cross-sectional epidermal profiles, the three gene modules exhibited consistent inter-module co-expression, with significant pairwise associations spanning circadian–oxidative stress, circadian–ferroptosis, and oxidative stress–ferroptosis axes. Although the correlation magnitudes were modest, the distributed associations suggest transcriptional links among these biological programs within the human epidermis. Granular analysis of individual candidates further yielded a specific mechanistic lead. Although *GPX4* did not act as a prominent cross-sectional co-expression hub with the circadian gene set, specific core clock genes—most notably *NR1D1*—displayed stronger co-expression with ferroptosis-related targets. Importantly, longitudinal analysis of paired epidermal samples from independent donors revealed that *NR1D1* expression exhibits positive temporal covariation with *GPX4*. While these observational transcriptomic data do not definitively establish direct transcriptional transactivation of *GPX4* by *NR1D1*, they identify a compelling circadian candidate whose temporal alignment with *GPX4* warranted functional validation. These findings therefore motivated us to examine whether modulation of clock-associated signaling alters cellular vulnerability to UVA-induced ferroptosis. Among the tested post-synchronization time points, UVA-associated injury was most pronounced at 24 h, as reflected by lower cell viability and GPX4 abundance and higher oxidative stress and lipid peroxidation. In contrast, intracellular Fe^2+^ levels did not differ significantly among time points within the UVA group, suggesting that temporal variation in UVA susceptibility was more closely associated with changes in oxidative and lipid-peroxidative responses than with differential Fe^2+^ accumulation. This pattern suggests that temporal variation in UVA susceptibility may reflect changes in the broader cellular state in which oxidative stress occurs rather than oscillation of a single ferroptosis effector. In this context, GPX4 may function as an important stress-responsive effector whose abundance and vulnerability vary with the temporal state of the cell.

The transcriptional response to UVA further supported this interpretation by revealing selective, gene-specific differences in temporal expression patterns. Specifically, UVA altered the temporal profiles of several clock genes, including *ARNTL*, *CLOCK*, and *PER2.* Notably, the temporal profiles of *NR1D1* and *RORA*, two components of the REV-ERB/ROR nuclear receptor network, showed heterogeneous responses to UVA exposure. *NR1D1* and *RORA* met the prespecified rhythmicity criterion in control cells but not following UVA exposure. These findings suggest that the temporal regulation of individual components within the REV-ERB/ROR network may be sensitive to UVA exposure, although the present data do not establish a significant between-group alteration in their rhythmic patterns. In parallel, redox- and ferroptosis-related genes also exhibited heterogeneous temporal profiles: *NFE2L2* met the rhythmicity criterion following UVA exposure, whereas *HMOX1*, *NQO1*, and *ALOX15* met the criterion only under control conditions. Thus, rather than indicating a uniform loss of rhythmicity, the observed changes point to gene-specific differences in the temporal organization of circadian and redox-related transcriptional programs.

The findings above led us to further consider *NR1D1*/REV-ERBα-associated signaling as a potential component of the temporal response to UVA. REV-ERBα has been implicated in the regulation of cellular redox homeostasis in a context-dependent manner. In retinal pigment epithelium, REV-ERBα supports NRF2-dependent antioxidant protection [20], whereas increased REV-ERB activity has been associated with ferroptotic injury in aristolochic-acid-induced renal injury, which was attenuated by the REV-ERB antagonist SR8278 [21]. These observations suggest that REV-ERBα-associated signaling may participate in cellular responses to oxidative stress and ferroptosis-associated injury, while its functional effects can vary across tissues and stress contexts. In the present study, pharmacological modulation of REV-ERBα with SR8278 partially preserved GPX4 abundance and cell viability following UVA exposure and reduced both oxidant accumulation and lipid peroxidation. Notably, these protective effects were accompanied by no significant change in Fe^2+^ accumulation, suggesting that REV-ERBα-associated signaling may be more closely related to the antioxidant and lipid-peroxidation response downstream of UVA-associated stress than to changes in intracellular labile iron. Together with the temporal and transcriptomic associations between *NR1D1* and *GPX4* expression, these findings suggest that *NR1D1*/REV-ERBα-associated signaling may be linked to the temporal regulation of GPX4-dependent antioxidant defense and, consequently, to variation in cellular susceptibility to UVA stress. Thus, the coordinated temporal changes in *NR1D1* and *GPX4* expression are consistent with a potential role of REV-ERBα-associated signaling in shaping the antioxidant response to UVA exposure. Previous studies in keratinocytes have indicated that the effects of SR8278 may vary with irradiation conditions and may involve REV-ERB-independent actions [22,23]. In the present study, the changes in GPX4 expression and cellular responses following SR8278 treatment are therefore consistent with a potential involvement of REV-ERB-associated signaling in the regulation of the antioxidant response to UVA, but should be considered within the broader pharmacological effects of SR8278. Accordingly, the present findings provide pharmacological support for an association between REV-ERBα-associated signaling and GPX4-dependent antioxidant defense, rather than establishing a REV-ERBα-specific regulatory effect on GPX4.

Several limitations of the present study should be considered. First, the experiments were conducted in synchronized proliferative HaCaT cells, which do not fully recapitulate the differentiated state of epidermal keratinocytes in vivo, at three irradiation time points. Although this experimental design enabled the identification of time-dependent patterns of UVA susceptibility, validation in primary keratinocytes or tissue-level skin models with denser temporal sampling will further strengthen the physiological relevance of these findings. Second, the transcriptomic analyses and pharmacological intervention provide convergent evidence linking temporal organization of clock-associated signaling to GPX4 preservation during UVA stress, while the precise molecular basis of this relationship remains to be established. The Kendall analysis based on four time points per donor provides a descriptive measure of temporal concordance but has limited power to characterize temporal dynamics. Future studies incorporating genetic and direct regulatory approaches will help further define the underlying mechanism.

Overall, the present study suggests that the temporal context in which keratinocytes encounter UVA may be an important determinant of their susceptibility to ferroptosis-related injury. Rather than indicating a simple circadian control of GPX4 itself, the findings support a broader model in which temporal organization of interconnected clock and redox processes may shape the capacity of GPX4-centered antioxidant defense to respond to UVA stress. Within this working model, *NR1D1*/REV-ERBα-associated signaling emerges as a candidate component of the clock–redox network that may influence the GPX4-centered response underlying this temporal susceptibility. This perspective extends the current understanding of UVA-associated cellular injury beyond dose-dependent responses and provides a basis for further defining how temporal cellular state influences antioxidant defense and stress vulnerability in the skin. Recent studies in skin-related models further highlight the broader relevance of interactions between circadian regulation and ferroptosis. Skin commensal-derived signaling has been shown to restore circadian rhythmicity in keratinocytes, suppress ferroptosis, and promote epidermal barrier repair in psoriasis, suggesting that the skin microenvironment may influence the clock–ferroptosis axis [24]. In a separate model of flap injury, disruption of circadian regulation was associated with rhythmic ferroptosis and impaired tissue viability, while stabilization of the core clock factor ARNTL attenuated ferroptosis and improved flap survival [25]. Together, these findings raise the possibility that temporal regulation of ferroptosis may extend beyond isolated keratinocyte responses to involve interactions among the molecular clock, redox homeostasis, and the broader skin microenvironment. Whether such mechanisms contribute to the temporal susceptibility of skin to UVA-induced injury remains to be determined.

## 4. Materials and Methods

### 4.1. Cell Culture

Human immortalized HaCaT keratinocytes (RRID:CVCL_0038) were obtained from the National Biomedical Experimental Cell Resource Bank, Cell Resource Center, Institute of Basic Medical Sciences, Chinese Academy of Medical Sciences (Beijing, China; Resource No. 3101HUMGNHu64). The cells were authenticated by STR profiling and tested negative for mycoplasma by the provider. Cells were cultured in MEM-α (Gibco, Thermo Fisher Scientific Inc., Waltham, MA, USA; C12571500BT) containing 10% fetal bovine serum (Gibco, Thermo Fisher Scientific Inc., Waltham, MA, USA; 100065998) and 1% penicillin–streptomycin (Cytiva, Marlborough, MA, USA; SV30010; 100 U/mL penicillin and 100 μg/mL streptomycin) at 37 °C and 5% CO_2_. Experiments used mycoplasma-negative cultures.

### 4.2. Cell Viability Assay

Cells were seeded at 5 × 10^3^ cells per well in 96-well plates. At 48 h post-treatment, 10 μL of CCK-8 reagent (Biosharp, Hefei Lanjieke Technology Co., Ltd., Hefei, China; BS350C) was added to each well and incubated for 1–2 h at 37 °C. Absorbance was read at 450 nm using a microplate reader (Molecular Devices, San Jose, CA, USA). Absorbance values from six technical replicates were averaged to yield a single value for each of the three independent biological experiments.

### 4.3. Cell Synchronization and Pharmacological Treatments

Confluent cells were synchronized with 100 nM dexamethasone (Sigma-Aldrich, St. Louis, MO, USA; D4902) in serum-free medium for 1 h, followed by a PBS wash and replacement with complete medium. The completion of the pulse and washout was designated as 0 h post-synchronization [4]. The 100 nM dexamethasone/1 h synchronization condition was selected in preliminary optimization experiments that varied dexamethasone concentration and/or exposure duration and assessed the subsequent temporal expression of the core clock genes ARNTL and CLOCK. This condition produced clear and reproducible temporal responses and was therefore used in the subsequent experiments. Prior to irradiation, cells were pretreated with ferrostatin-1 (Fer-1; 5 μM, 1 h; Selleck Chemicals, Houston, TX, USA; Cat. No. S7243), MG132 (10 μM, 12 h; MedChemExpress, Monmouth Junction, NJ, USA; Cat. No. HY-13259), or SR8278 (10 μM, 12 h; Merck/Calbiochem, Darmstadt, Germany; Cat. No. 554718). The Fer-1 concentration (5 μM) was selected based on preliminary cytotoxicity evaluations (Figure S1C). Vehicle control groups received an equivalent concentration of DMSO (final concentration ≤ 0.1%, *v*/*v*). All compounds remained in the culture medium post-irradiation.

### 4.4. Ultraviolet Irradiation

Cells were washed twice with pre-warmed PBS and irradiated in PBS under UVA or UVB sources. UVA was delivered using a long-wave ultraviolet LED lamp (ZLUVLAMP, Shenzhen, China;HU287; nominal wavelength, 365 nm) at a source-to-sample distance of 15 cm and an irradiance of 30 mW/cm^2^. UVB was supplied by two fluorescent lamps (model UVB F8T5; purchase identifier CZ356; nominal emission range, 300–312 nm) at a distance of 10 cm and an irradiance of 1 mW/cm^2^. Irradiance at the sample plane was quantified using a calibrated UV radiometer (Xinbao Scientific Instruments, Shenzhen, China; HX8303A). The standard doses used in subsequent experiments were 10 J/cm^2^ for UVA and 5 mJ/cm^2^ for UVB, corresponding to exposure durations of approximately 333 s (5.6 min) and 5 s, respectively. These working doses were selected based on preliminary dose–response experiments, taking into consideration cellular viability and the relevance of the exposure levels to reported human UV exposure. Exposure times were calculated as dose (J/cm^2^) divided by irradiance (W/cm^2^). To prevent heat accumulation during irradiation, culture plates were kept on an aluminum cold block. Immediately after irradiation, PBS was replaced with complete medium with or without indicated compounds. Non-irradiated control cells underwent identical procedures without UV exposure.

For the transcriptional time-series experiments, synchronized cells were irradiated once with UVA at 0 h post-synchronization and subsequently sampled at 6 h intervals through 48 h. For experiments assessing the effect of the timing of UVA exposure, cells were first synchronized as described above and subsequently irradiated with UVA at 4, 12, or 24 h post-synchronization.

### 4.5. Oxidant-Sensitive DCFH-DA Fluorescence Assay

At 1 h post-irradiation, cells were loaded with 10 μM DCFH-DA (Solarbio, Beijing, China; CA1410) in serum-free medium for 30 min at 37 °C in the dark. After three washes with PBS, cellular fluorescence (excitation/emission: 488/525 nm) was captured under identical acquisition parameters across all experimental groups. Mean fluorescence intensity was calculated from five randomly selected non-overlapping fields per condition using ImageJ software (version 1.54). Field-level values were averaged to represent each independent experiment (*n* = 3).

### 4.6. Lipid Peroxidation Assessment by Liperfluo

At 4 h post-irradiation, cells were incubated with 2 μM Liperfluo (Dojindo Laboratories, Kumamoto, Japan; L248) for 30 min at 37 °C in the dark. After washing thrice with PBS, green fluorescence (excitation/emission: 488/535 nm) was imaged under constant exposure conditions. Fluorescence intensity was quantified across five random fields per group via ImageJ, and averaged values were derived from three independent biological replicates.

### 4.7. Intracellular Fe^2+^ Determination

At 2 h post-irradiation, intracellular Fe^2+^ levels were measured using a colorimetric assay kit (Solarbio, Beijing, China; BC5415) according to the manufacturer’s protocols. Absorbance was recorded at 593 nm and normalized to total protein concentration determined by a BCA assay. Technical duplicates/triplicates were averaged for each of the three independent experiments.

### 4.8. Quantitative Real-Time PCR

Total RNA was extracted using TRIzol Reagent (Invitrogen, Thermo Fisher Scientific Inc., Waltham, MA, USA;15596026) according to the manufacturer’s instructions. One microgram of total RNA was reverse-transcribed using the ReverTra Ace qPCR RT Master Mix with gDNA Remover (Toyobo, Osaka, Japan; FSQ-301). Quantitative PCR was performed with the FastReal SYBR Green Mix (Tiangen Biotech, Beijing, China; FP217) on an Applied Biosystems 7300Plus Real-Time PCR System under the following cycling conditions: initial denaturation at 95 °C for 30 s, followed by 40 cycles of 95 °C for 5 s and 60 °C for 30 s. Target gene expression was normalized to *RPL13A* as the internal reference, which showed generally consistent expression across the experimental conditions examined. Relative gene expression levels were calculated using the 2^−ΔΔCt^ method or represented as −ΔCt/−ΔΔCt values for cosinor and dynamic interaction modeling, as specified in the corresponding figure legends. Primer sequences used in this study are detailed in Supplementary Table S1.

### 4.9. Western Blotting

Cells were harvested and lysed in NETN100 buffer supplemented with a 1× cOmplete Protease Inhibitor Cocktail (Roche Diagnostics, Mannheim, Germany; Cat. No. 11836170001). Protein concentrations were determined using a BCA assay kit. Equal amounts of protein (40 μg per lane) were resolved on 10% SDS-PAGE gels and transferred onto nitrocellulose membranes. Membranes were blocked with 5% non-fat milk in TBST for 1.5 h at room temperature and subsequently incubated overnight at 4 °C with primary antibodies against BMAL1 (Signalway Antibody [SAB], Greenbelt, MD, USA;21415), GPX4 (SAB, Greenbelt, MD, USA;29146), xCT (SAB, Greenbelt, MD, USA; 62016), REV-ERBα (SAB, Greenbelt, MD, USA; 36659), and GAPDH (Affinity Biosciences, Cincinnati, OH, USA; AF7021), each diluted 1:1000. After washing, membranes were incubated with HRP-conjugated goat anti-rabbit IgG secondary antibody (Abbkine, Wuhan, China; A25022; 1:4000) for 1 h at room temperature. Protein bands were visualized using an ECL substrate (Biosharp, Hefei Lanjieke Technology Co., Ltd., Hefei, China; BL523B) and captured within the linear range using a Tanon-5200 Chemiluminescent Imaging System. Densitometric quantification was performed using ImageJ software, and target protein abundance was normalized to GAPDH across three independent biological replicates (*n* = 3), with retrospective assessment showing no significant condition-dependent variation in GAPDH signal.

### 4.10. Fixed-Period Cosinor and Time-Series Analysis

To characterize baseline temporal rhythmicity, synchronized cells without UV irradiation were collected every 6 h over a 48 h period for gene expression profiling. For UVA time-course studies, synchronized cells received UVA irradiation (10 J/cm^2^) at 0 h post-synchronization and were harvested every 6 h up to 48 h.

Gene expression temporal rhythmicity was assessed using −ΔCt values with a linear cosinor model incorporating fixed 24 h cosine and sine components and an experimental-batch covariate. A 24 h period was fixed a priori because the analysis targeted temporal variation on the canonical mammalian circadian timescale, and sampling every 6 h over 48 h provided observations spanning two nominal 24 h cycles. The linear cosinor model assumes an approximately sinusoidal waveform with approximately normal and homoscedastic residuals. Within-group rhythmicity was assessed by jointly testing the cosine and sine coefficients against a reduced model without the periodic terms. Batch-adjusted mesor, amplitude, and acrophase were derived from the fitted cosinor model. Between-group differences in circadian patterns were evaluated using linear models incorporating treatment group, cosine and sine terms, and treatment-by-cosinor interaction terms (Group × cos(t) and Group × sin(t)). Group-specific fitted curves and means ± SE were transformed back to the plotting scale for visualization. To account for multiple testing across prespecified candidate genes, the Benjamini–Hochberg procedure was applied separately to within-group rhythmicity tests and between-group cosinor interaction tests [26]. An FDR-adjusted q < 0.05 was considered statistically significant for within-group rhythmicity or a difference in circadian waveform between groups. All cosinor modeling and time-series analyses were performed using R software (version 4.4.2).

### 4.11. Human Skin Transcriptomic Data Processing and Co-Expression Network Analysis

Processed log_2_ expression matrices and clinical metadata were obtained from the GEO database (GSE112660 [27] and GSE139301 [28]; GPL13667 platform). Sample accessions, demographics, body sites, and sampling time points were standardized. These datasets consisted of publicly available human epidermal transcriptomic profiles rather than experimentally generated skin explants; donor characteristics were reported only to the extent available in the original GEO metadata. Microarray probes were mapped to official gene symbols, and multiple probes mapping to the same gene were averaged per sample.

For cross-sectional population co-expression analysis, 219 cross-sectional forearm epidermal profiles from independent donors in GSE112660 (152 European and 67 African ancestry) were evaluated. Prespecified gene modules for ferroptosis, oxidative stress, and circadian clock pathways were compiled from FerrDb V2, MSigDB, and the Reactome Circadian Clock pathway, respectively (Supplementary Table S2). Between-module relationships were assessed using pairwise Spearman rank correlations between genes belonging to each pair of functional gene sets. For each module pair, the mean absolute Spearman correlation coefficient (mean |ρ|) was calculated to summarize the overall strength of gene-level associations, while the mean signed Spearman correlation coefficient was used to represent the overall direction of these associations. These values were visualized as edges connecting the three functional modules, with edge width and labels representing mean |ρ| and edge color representing the mean signed correlation coefficient (Figure 3B).

To quantify inter-module gene coupling, pairwise Spearman rank correlation coefficients (ρ) and corresponding *p* values were calculated using the Hmisc package (rcorr function), with Benjamini–Hochberg false discovery rate (FDR) correction applied (q < 0.05). The coupling strength of a specific gene relative to a target module was quantified as the mean of the absolute Spearman correlation coefficients (mean |ρ|) between that gene and all constituent genes within the target module. Ranked horizontal bar plots were generated based on mean |ρ| values to highlight top-coupled driver candidates between ferroptosis/oxidative stress and core circadian modules. Given the limited donor-level demographic information available in the dataset, no covariate adjustment was performed. The transcriptomic analysis was therefore conducted as an unadjusted descriptive correlation analysis.

For longitudinal temporal analysis, longitudinal epidermal transcriptomes (GSE139301) collected at 00:00, 06:00, 12:00, and 18:00 from 19 healthy male donors (*n* = 19; 76 total profiles) were analyzed. To capture monotonic intra-individual trajectory concordance while accommodating non-linear dynamics, non-parametric Kendall’s rank correlation coefficients (τ) were computed between *GPX4* and target candidate genes across the four time points independently for each donor. Given the four time points available per donor, Kendall’s τ was used as a descriptive measure of monotonic temporal concordance rather than as a high-resolution characterization of temporal dynamics.

For each candidate gene across the 19 donors, donor-level τ distributions were summarized by mean τ, mean |τ|, and exact two-sided Wilcoxon signed-rank tests against zero with Benjamini–Hochberg FDR correction q < 0.05. Nine key candidates across circadian, ferroptosis, and oxidative stress modules were prioritized, and their donor-level τ distributions were visualized using violin plots to assess inter-individual consistency and temporal coupling stability. Medians and interquartile ranges were reported to summarize the donor-level distributions shown in Figure 3E. All transcriptomic processing and statistical bioinformatics analyses were performed using R (version 4.4.2).

### 4.12. Statistical Analysis

Wet-laboratory experiments were independently performed in three biological replicates (*n* = 3), with technical replicates averaged within each independent experiment and not treated as independent observations. Data are presented as mean ± SD, except for time-series cosinor profiles, which are presented as mean ± SE. Ordinary one-way ANOVA was used for single-factor comparisons in Figure 1C–E, Figure 2B,F,H, Figure 7A,C,E,G,H and Figure S1A–C. For Figure 1F–H and Figure S1D, ordinary two-way ANOVA was performed with UV condition and Fer-1 treatment as factors. For Figure 5, ordinary two-way ANOVA was performed with post-synchronization time and treatment as factors, and the time × treatment interaction was evaluated [29]. Prespecified pairwise comparisons following ANOVA were adjusted using the Šídák procedure. For the RT-qPCR time courses in Figure 2C,D,I–K, −ΔCt values were analyzed using repeated-measures linear models containing wavelength, time, and their interaction as fixed effects, with experimental batch treated as the repeated-measures subject. The resulting interaction *p* values were adjusted across the five prespecified genes using the Benjamini–Hochberg procedure. Figure 5 interaction *p* values were similarly adjusted across the five prespecified endpoints. Fixed-period cosinor and transcriptomic analyses are described in Section 4.10 and Section 4.11, respectively. An adjusted *p* or FDR-adjusted q value < 0.05 was considered statistically significant. *p* values below 0.0001 are reported as *p* < 0.0001. Wet-laboratory analyses were performed using GraphPad Prism version 11.0.0, whereas cosinor and transcriptomic analyses were performed using R version 4.4.2. Exact adjusted *p* values for prespecified pairwise comparisons are provided in Supplementary Table S4.

## Data Availability

The original contributions presented in this study are included in the article/supplementary material. Further inquiries can be directed to the corresponding author(s).

## Supplementary Materials

The following supporting information can be downloaded at: https://www.mdpi.com/article/10.3390/ijms27208947/s1.

## Abbreviations

**Abbreviation**	**Full name**
ACSL4	acyl-CoA synthetase long-chain family member 4
ALOX15	arachidonate 15-lipoxygenase
ARNTL/BMAL1	aryl hydrocarbon receptor nuclear translocator-like protein 1/brain and muscle ARNT-like protein 1
CAT	catalase
CLOCK	circadian locomotor output cycles protein kaput
CRY2	cryptochrome circadian regulator 2
FTH1	ferritin heavy chain 1
GAPDH	glyceraldehyde-3-phosphate dehydrogenase
GPX4	glutathione peroxidase 4
HMOX1	heme oxygenase 1
NFE2L2/NRF2	nuclear factor erythroid 2-related factor 2
NQO1	NAD(P)H quinone dehydrogenase 1
NR1D1/REV-ERBα	nuclear receptor subfamily 1 group D member 1/REV-ERBα
NR1D2	nuclear receptor subfamily 1 group D member 2
PER2	period circadian regulator 2
RORA	RAR-related orphan receptor alpha
RPL13A	ribosomal protein L13a
SAT1	spermidine/spermine N1-acetyltransferase 1
SLC7A11/xCT	solute carrier family 7 member 11/cystine/glutamate antiporter system Xc− light chain
SOD2	superoxide dismutase 2
TFRC	transferrin receptor
