# Supplementary material for: Temporal Regulation of UVA-Induced Ferroptosis-Associated Injury in Keratinocytes
Source: Int J Mol Sci. 2026 Oct 9;27(20):8947. doi: 10.3390/ijms27208947 (PMC13704125; doi:10.3390/ijms27208947)
Supplement: Supplementary file 1 [file ijms-27-08947-s001.zip › Supplementary Figure legends.pdf]

Supplementary Figure legends

Supplementary Figure S1. Dose-dependent effects of UVA and UVB on HaCaT cell viability and intracellular Fe^{2+} levels and effects of ferrostatin-1 treatment.

(A, B) HaCaT cell viability at 48 h following graded UVA or UVB irradiation. (C) HaCaT cell viability at 48 h following Fer-1 treatment (0–10 μM). (D) Intracellular Fe^{2+} levels at 2 h following UVA (10 J/cm²) or UVB (5 mJ/cm²) irradiation with or without Fer-1 (5 μM). Data are presented as mean ± SD from three independent experiments (n = 3). For A − C, statistical significance was assessed by ordinary one-way ANOVA followed by Šídák-adjusted prespecified comparisons. For D, ordinary two-way ANOVA with UV condition and Fer-1 treatment as factors was used, followed by Šídák-adjusted prespecified comparisons. *P < 0.05 versus the matched control; #P < 0.05 versus the matched UV-only group.

Supplementary Figure S2. Additional clock-gene expression profiles following UVA exposure.

Synchronized HaCaT cells were irradiated with UVA (10 J/cm²) at 0 h post-synchronization and sampled every 6 h through 48 h. *CLOCK* (A), *RORA* (B), *PER2* (C), and *CRY2* (D) were measured every 6 h through 48 h following UVA exposure. Data are presented as mean ± SE from three independent batches (n = 3), and values are displayed as −ΔΔCt. Models were fitted to −ΔCt. Solid curves show significant 24-h cosinor fits; dashed lines connect the observed means when rhythmicity did not meet the significance criterion. P values indicate group × 24-h cosinor interaction effects, and FDR-adjusted q values were calculated across the 10 prespecified genes in Figure 6 and Supplementary Figure S2 using the Benjamini–Hochberg procedure.
