# Supplementary material for: Temporal Regulation of UVA-Induced Ferroptosis-Associated Injury in Keratinocytes
Source: Int J Mol Sci. 2026 Oct 9;27(20):8947. doi: 10.3390/ijms27208947 (PMC13704125; doi:10.3390/ijms27208947)
Supplement: Supplementary file 1 [file ijms-27-08947-s001.zip › Supplementary Figure S1.pdf]

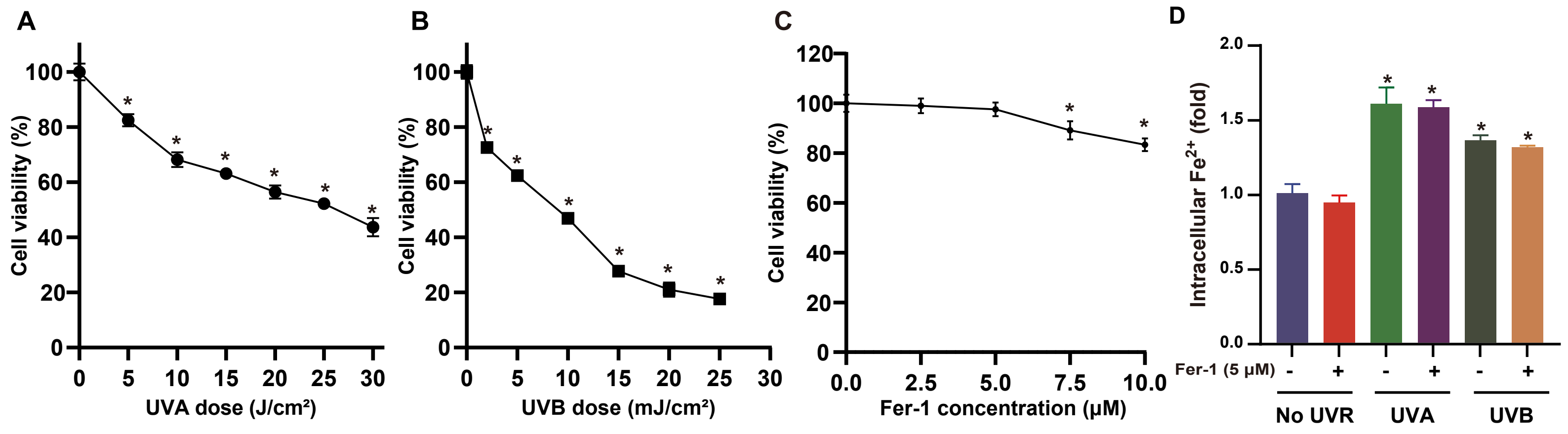
A
Cell viability (%)
UVA dose (J/cm²)
B
Cell viability (%)
UVB dose (mJ/cm²)
C
Cell viability (%)
Fer-1 concentration (μM)
D
Intracellular Fe2+ (fold)
Fer-1 (5 μM)
No UVR
UVA
UVB
