# Supplementary material for: Temporal Regulation of UVA-Induced Ferroptosis-Associated Injury in Keratinocytes
Source: Int J Mol Sci. 2026 Oct 9;27(20):8947. doi: 10.3390/ijms27208947 (PMC13704125; doi:10.3390/ijms27208947)
Supplement: Supplementary file 1 [file ijms-27-08947-s001.zip › Supplementary Figure S2.pdf]

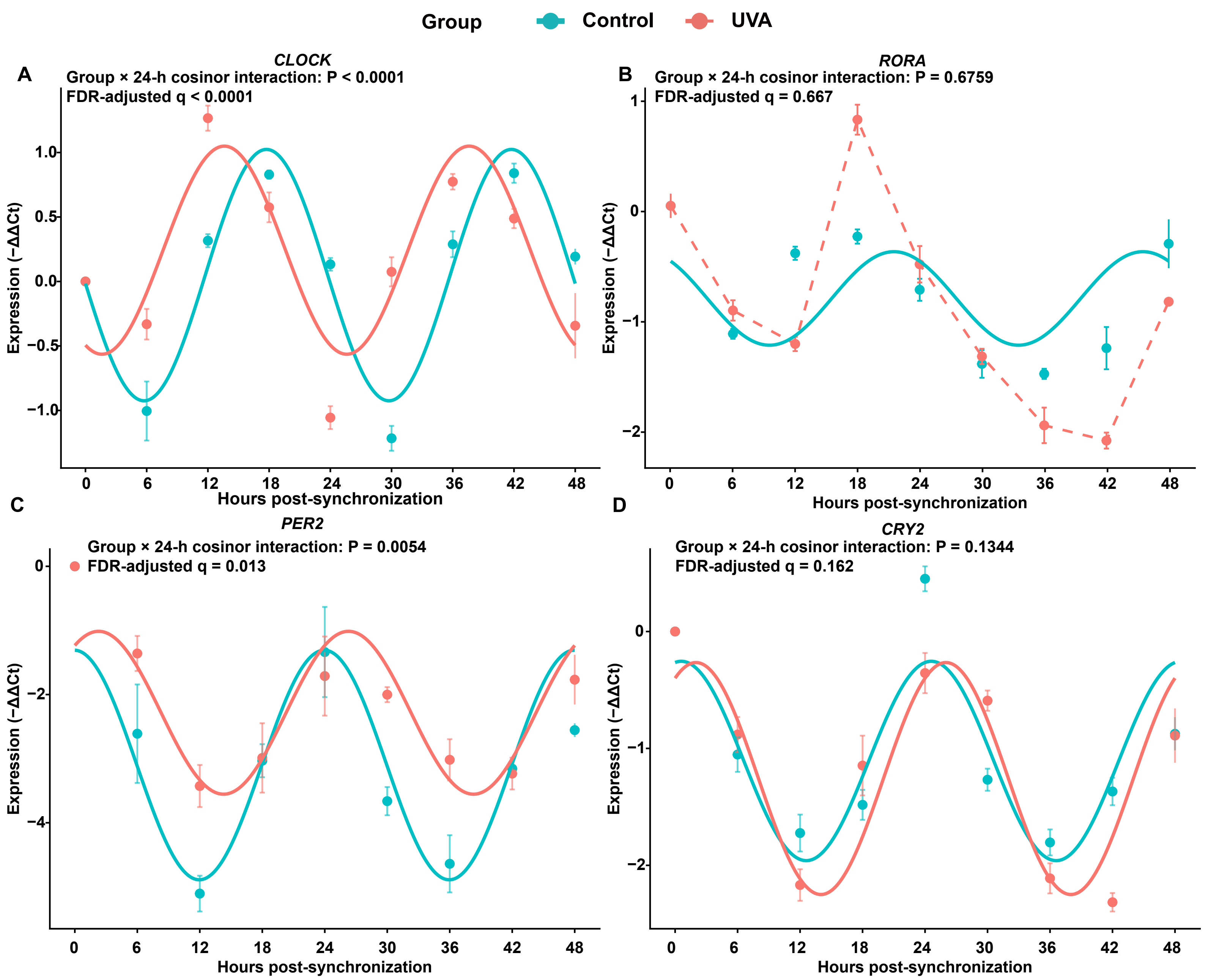

Group
Control
UVA
A
CLOCK
Group × 24-h cosinor interaction: P < 0.0001
FDR-adjusted q < 0.0001
B
RORA
Group × 24-h cosinor interaction: P = 0.6759
FDR-adjusted q = 0.667
C
PER2
Group × 24-h cosinor interaction: P = 0.0054
FDR-adjusted q = 0.013
D
CRY2
Group × 24-h cosinor interaction: P = 0.1344
FDR-adjusted q = 0.162
Expression (−ΔΔCt)
Hours post-synchronization
0
6
12
18
24
30
36
42
48
