# Supplementary material for: Temporal Regulation of UVA-Induced Ferroptosis-Associated Injury in Keratinocytes
Source: Int J Mol Sci. 2026 Oct 9;27(20):8947. doi: 10.3390/ijms27208947 (PMC13704125; doi:10.3390/ijms27208947)
Supplement: Supplementary file 1 [file ijms-27-08947-s001.zip › Supplementary Table S1.pdf]

Supplementary Table S1. Primer sequences used for quantitative real-time PCR (RT-qPCR).

Gene	Forward primer (5′–3′)	Reverse primer (5′–3′)
ARNTL	AAGACTTCCCCTCTACCTGCTC	AACTACATGAGAATGCAGTCGTC
GPX4	CCCGATACGCTGAGTGTGGTTTG	TCTTCGTTACTCCCTGGCTCCTG
SLC7A11	ACGGTGGTGTGTTTGCTGTCTC	GCTGGTAGAGGAGTGTGCTTGC
PER2	TTGGACAGCGTCATCAGGTA	TCCGCTTATCACTGGACCTT
CLOCK	CAGCCAGTGATGTCTCAAGC	ATGCGTGTCCGTTGTTCC
NFE2L2	TCCAGTCAGAAACCAGTGGAT	GAATGTCTGCGCCAAAAGCTG
HMOX1	AGGCCAAGACTGCGTTCCT	GGTGTAAGGACCCATCGGAG
NQO1	AGGCTGGTTTGAGCGAGTTA	TGATGGGATTGAAGTTCATGGC
SOD2	CTGGACAAACCTCAGCCCTAAC	AACCTGAGCCTTGGACACCAAC
CAT	CGTGCTGAATGAGGAACAG	AGTCAGGGTGGACGTCAGTG
RPL13A	CCTGGAGGAGAAGAGGAAAGAGA	TTGAGGACCTCTGTGTATTTGTCAA
RORA	CTTGCCGTAGGGATGTCTCG	GAAGTTCCGTCAGCCCGTT
ALOX15	TCAGGTTCCCTTGTTACCGC	CAGCTCTTCTTCCCGGTGTT
NR1D1	CGAACTCTGCACTTCTCTCTC	GGAGGGAGGCAGGTATTTACA
CRY2	CTGTTCAAGGAATGGGGAGTG	GGTCATAGAGGGTATGAGAATTC
