# Supplementary material for: Temporal Regulation of UVA-Induced Ferroptosis-Associated Injury in Keratinocytes
Source: Int J Mol Sci. 2026 Oct 9;27(20):8947. doi: 10.3390/ijms27208947 (PMC13704125; doi:10.3390/ijms27208947)
Supplement: Supplementary file 1 [file ijms-27-08947-s001.zip › Supplementary Table S2.pdf]

Supplementary Table S2. Prespecified gene sets used for co-expression analyses of public human epidermal transcriptomes.

Module	Gene symbol	Gene name	Functional category
Circadian rhythm	*ARNTL*	Aryl hydrocarbon receptor nuclear translocator-like protein 1	Core clock
Circadian rhythm	*CLOCK*	Circadian locomotor output cycles protein kaput	Core clock
Circadian rhythm	*PER1*	Period circadian regulator 1	Clock-controlled
Circadian rhythm	*PER2*	Period circadian regulator 2	Clock-controlled
Circadian rhythm	*PER3*	Period circadian regulator 3	Clock-controlled
Circadian rhythm	*CRY1*	Cryptochrome circadian regulator 1	Core clock
Circadian rhythm	*CRY2*	Cryptochrome circadian regulator 2	Core clock
Circadian rhythm	*NR1D1*	Nuclear receptor subfamily 1 group D member 1	Clock nuclear receptor
Circadian rhythm	*NR1D2*	Nuclear receptor subfamily 1 group D member 2	Clock nuclear receptor
Circadian rhythm	*RORA*	RAR-related orphan receptor A	Clock nuclear receptor
Circadian rhythm	*RORC*	RAR-related orphan receptor C	Clock nuclear receptor
Circadian rhythm	*DBP*	D-site albumin promoter binding protein	Clock output gene
Circadian rhythm	*TEF*	Thyrotroph embryonic factor	Clock output gene
Circadian rhythm	*HLF*	Hepatic leukemia factor	Clock output gene
Circadian rhythm	*NFIL3*	Nuclear factor interleukin-3 regulated	Clock output gene
Oxidative stress	*NFE2L2*	Nuclear factor erythroid 2-related factor 2	Antioxidant defense
Oxidative stress	*NQO1*	NAD(P)H quinone dehydrogenase 1	Nrf2 downstream antioxidant
Oxidative stress	*HMOX1*	Heme oxygenase 1	Nrf2 downstream antioxidant
Oxidative stress	*SOD1*	Superoxide dismutase 1	ROS scavenging

Module	Gene symbol	Gene name	Functional category
Oxidative stress	*SOD2*	Superoxide dismutase 2	ROS scavenging
Oxidative stress	*CAT*	Catalase	ROS scavenging
Oxidative stress	*GPX1*	Glutathione peroxidase 1	Glutathione-dependent antioxidant
Oxidative stress	*PRDX1*	Peroxiredoxin 1	Peroxiredoxin family
Oxidative stress	*PRDX2*	Peroxiredoxin 2	Peroxiredoxin family
Oxidative stress	*PRDX3*	Peroxiredoxin 3	Peroxiredoxin family
Oxidative stress	*PRDX4*	Peroxiredoxin 4	Peroxiredoxin family
Oxidative stress	*PRDX5*	Peroxiredoxin 5	Peroxiredoxin family
Oxidative stress	*PRDX6*	Peroxiredoxin 6	Peroxiredoxin family
Oxidative stress	*TXN*	Thioredoxin	Thioredoxin system
Oxidative stress	*TXNRD1*	Thioredoxin reductase 1	Thioredoxin system
Oxidative stress	*GSR*	Glutathione reductase	Glutathione recycling
Oxidative stress	*GCLC*	Glutamate-cysteine ligase catalytic subunit	Glutathione synthesis
Oxidative stress	*GCLM*	Glutamate-cysteine ligase regulatory subunit	Glutathione synthesis
Ferroptosis-related	*GPX4*	Glutathione peroxidase 4	Ferroptosis defense
Ferroptosis-related	*SLC7A11*	Solute carrier family 7 member 11	Cystine import / ferroptosis defense
Ferroptosis-related	*ACSL4*	Acyl-CoA synthetase long-chain family member 4	Polyunsaturated fatty acid activation
Ferroptosis-related	*LPCAT3*	Lysophosphatidylcholine acyltransferase 3	Membrane PUFA remodeling
Ferroptosis-related	*ALOX15*	Arachidonate 15-lipoxygenase	Lipid peroxidation execution
Ferroptosis-related	*ALOX5*	Arachidonate 5-lipoxygenase	Lipid peroxidation execution
Ferroptosis-related	*TFRC*	Transferrin receptor	Iron uptake

Module	Gene symbol	Gene name	Functional category
Ferroptosis-related	*FTH1*	Ferritin heavy chain 1	Iron storage
Ferroptosis-related	*FTL*	Ferritin light chain	Iron storage
Ferroptosis-related	*NCOA4*	Nuclear receptor coactivator 4	Ferritinophagy / iron release
Ferroptosis-related	*SAT1*	Spermidine/spermine N1-acetyltransferase 1	Polyamine metabolism-ferroptosis regulator

Note: The author-curated gene sets were prespecified for this analysis on the basis of canonical circadian, oxidative-stress, and ferroptosis-related functions; the complete operational lists are reported here.
