# Supplementary material for: Temporal Regulation of UVA-Induced Ferroptosis-Associated Injury in Keratinocytes
Source: Int J Mol Sci. 2026 Oct 9;27(20):8947. doi: 10.3390/ijms27208947 (PMC13704125; doi:10.3390/ijms27208947)
Supplement: Supplementary file 1 [file ijms-27-08947-s001.zip › Supplementary Table S3.pdf]

Supplementary Table S3. Circadian rhythmicity and differences in circadian patterns of clock- and oxidative stress-related genes between Control and UVA groups.

Gene	Group	Mesor (−ΔCt)	Amplitude	Acrophase (h post-synchronization)	q_{rhythm}	$q_{UVA\times rhythm}$
ARNTL	Control	−6.312	0.783	10.9	<0.001	**0.002**
	UVA	−7.405	1.286	12.9	<0.001	
NR1D1	Control	−4.353	0.936	21.9	0.001	**0.259**
	UVA	−4.425	0.451	21.9	0.136	
NFE2L2	Control	−4.061	0.429	21.8	0.17	**0.02**
	UVA	−4.522	0.668	6.3	0.042	
HMOX1	Control	−13.082	1.241	8.3	0.008	**0.162**
	UVA	−15.578	0.432	7.3	0.195	
NQO1	Control	−5.503	0.328	0.6	0.003	**0.162**
	UVA	−5.752	0.019	5.5	0.992	
ALOX15	Control	−14.755	0.501	21.4	<0.001	**0.01**
	UVA	−14.631	0.114	0.1	0.388	
CLOCK	Control	−8.546	0.541	18	<0.001	**<0.001**
	UVA	−9.365	0.807	13.6	<0.001	
RORA	Control	−11.754	0.401	21.5	0.033	**0.667**
	UVA	−11.676	0.582	22.5	0.051	
PER2	Control	−9.980	1.79	0	<0.001	**0.013**
	UVA	−10.095	1.27	2.3	<0.001	
CRY2	Control	−14.308	0.853	0.6	<0.001	**0.162**
	UVA	−15.600	0.992	2	<0.001	

Mesor, amplitude, and acrophase were estimated using a batch-adjusted 24-h cosinor model and are presented on the −ΔCt scale (acrophase in hours after synchronization). q_{rhythm} and $q_{UVA\times rhythm}$ denote Benjamini–Hochberg FDR-adjusted P values for circadian rhythm presence within each group and for waveform differences between groups, respectively. $q < 0.05$ was considered statistically significant.
