# Supplementary material for: Temporal Regulation of UVA-Induced Ferroptosis-Associated Injury in Keratinocytes
Source: Int J Mol Sci. 2026 Oct 9;27(20):8947. doi: 10.3390/ijms27208947 (PMC13704125; doi:10.3390/ijms27208947)
Supplement: Supplementary file 1 [file ijms-27-08947-s001.zip › Supplementary Table S4.pdf]

Supplementary Table S4. Adjusted P values for prespecified pairwise comparisons supporting figure annotations and result statements.

Figure	Panel	Endpoint	Comparison	Statistical test / adjustment	Adjusted P
Figure 1	C	DCFH-DA	UVA (5 J/cm^2) vs Control	Ordinary one-way ANOVA; Šídák-adjusted prespecified comparisons	0.00049251
Figure 1	C	DCFH-DA	UVA (10 J/cm^2) vs Control	Ordinary one-way ANOVA; Šídák-adjusted prespecified comparisons	5.66724E-09
Figure 1	C	DCFH-DA	UVB(2 mJ/cm^2) vs Control	Ordinary one-way ANOVA; Šídák-adjusted prespecified comparisons	0.000563391
Figure 1	C	DCFH-DA	UVB (5 mJ/cm^2) vs Control	Ordinary one-way ANOVA; Šídák-adjusted prespecified comparisons	2.95545E-08
Figure 1	D	Liperfluo	UVA(5 J/cm^2) vs Control	Ordinary one-way ANOVA; Šídák-adjusted prespecified comparisons	5.21835E-10
Figure 1	D	Liperfluo	UVA(10 J/cm^2) vs Control	Ordinary one-way ANOVA; Šídák-adjusted prespecified comparisons	3.61666E-12
Figure 1	D	Liperfluo	UVB (2 mJ/cm^2) vs Control	Ordinary one-way ANOVA; Šídák-adjusted prespecified comparisons	4.01367E-10
Figure 1	D	Liperfluo	UVB(5 mJ/cm^2) vs Control	Ordinary one-way ANOVA; Šídák-adjusted prespecified comparisons	1.52367E-12
Figure 1	E	Fe^{2+}	UVA (5 J/cm^2) vs Control	Ordinary one-way ANOVA; Šídák-adjusted prespecified comparisons	0.002084323
Figure 1	E	Fe^{2+}	UVA (10 J/cm^2) vs Control	Ordinary one-way ANOVA; Šídák-adjusted prespecified comparisons	4.97262E-08
Figure 1	E	Fe^{2+}	UVB (2 mJ/cm^2) vs Control	Ordinary one-way ANOVA; Šídák-adjusted prespecified comparisons	0.006037938
Figure 1	E	Fe^{2+}	UVB (5 mJ/cm^2) vs Control	Ordinary one-way ANOVA; Šídák-adjusted prespecified comparisons	7.51634E-07
Figure 1	F	Viability	UVA vs Control	Ordinary two-way ANOVA (UV condition × Fer-1); Šídák-adjusted prespecified comparisons	2.52215E-09
Figure 1	F	Viability	UVB vs Control	Ordinary two-way ANOVA (UV condition × Fer-1); Šídák-adjusted prespecified comparisons	2.88338E-10
Figure 1	F	Viability	UVA+Fer-1 vs UVA	Ordinary two-way ANOVA (UV condition × Fer-1); Šídák-adjusted prespecified comparisons	1.00824E-07
Figure 1	F	Viability	UVB+Fer-1 vs UVB	Ordinary two-way ANOVA (UV condition × Fer-1); Šídák-adjusted prespecified comparisons	2.39234E-07
Figure 1	G	DCFH-DA	UVA vs Control	Ordinary two-way ANOVA (UV condition × Fer-1); Šídák-adjusted prespecified comparisons	1.30883E-09

Figure	Panel	Endpoint	Comparison	Statistical test / adjustment	Adjusted P
Figure 1	G	DCFH-DA	UVB vs Control	Ordinary two-way ANOVA (UV condition × Fer-1); Šídák-adjusted prespecified comparisons	9.63975E-09
Figure 1	G	DCFH-DA	UVA+Fer-1 vs UVA	Ordinary two-way ANOVA (UV condition × Fer-1); Šídák-adjusted prespecified comparisons	6.1414E-08
Figure 1	G	DCFH-DA	UVB+Fer-1 vs UVB	Ordinary two-way ANOVA (UV condition × Fer-1); Šídák-adjusted prespecified comparisons	5.72083E-09
Figure 1	H	Liperfluo	UVA vs Control	Ordinary two-way ANOVA (UV condition × Fer-1); Šídák-adjusted prespecified comparisons	1.05476E-11
Figure 1	H	Liperfluo	UVB vs Control	Ordinary two-way ANOVA (UV condition × Fer-1); Šídák-adjusted prespecified comparisons	2.09344E-12
Figure 1	H	Liperfluo	UVA+Fer-1 vs UVA	Ordinary two-way ANOVA (UV condition × Fer-1); Šídák-adjusted prespecified comparisons	0.000180596
Figure 1	H	Liperfluo	UVB+Fer-1 vs UVB	Ordinary two-way ANOVA (UV condition × Fer-1); Šídák-adjusted prespecified comparisons	3.32423E-05
Figure 2	B	GPX4	UVA1 vs Control	Ordinary one-way ANOVA; Šídák-adjusted prespecified comparisons	0.001121327
Figure 2	B	GPX4	UVA2 vs Control	Ordinary one-way ANOVA; Šídák-adjusted prespecified comparisons	5.42279E-05
Figure 2	B	GPX4	UVB1 vs Control	Ordinary one-way ANOVA; Šídák-adjusted prespecified comparisons	7.38048E-05
Figure 2	B	GPX4	UVB2 vs Control	Ordinary one-way ANOVA; Šídák-adjusted prespecified comparisons	6.65836E-07
Figure 2	F	xCT	UVB vs Control	Ordinary one-way ANOVA; Šídák-adjusted prespecified comparisons	7.87897E-08
Figure 2	F	xCT	UVB+Fer-1 vs UVB	Ordinary one-way ANOVA; Šídák-adjusted prespecified comparisons	0.000103027
Figure 2	F	GPX4	UVA vs Control	Ordinary one-way ANOVA; Šídák-adjusted prespecified comparisons	1.40306E-09
Figure 2	F	GPX4	UVA+Fer-1 vs UVA	Ordinary one-way ANOVA; Šídák-adjusted prespecified comparisons	0.000296816
Figure 2	F	GPX4	UVB vs Control	Ordinary one-way ANOVA; Šídák-adjusted prespecified comparisons	4.76112E-11
Figure 2	F	GPX4	UVB+Fer-1 vs UVB	Ordinary one-way ANOVA; Šídák-adjusted prespecified comparisons	0.000337906

Figure	Panel	Endpoint	Comparison	Statistical test / adjustment	Adjusted P
Figure 5	A	Viability	UVA 4 h vs Control 4 h	Ordinary two-way ANOVA (time × treatment); Šídák-adjusted prespecified comparisons	1.8221E-11
Figure 5	A	Viability	UVA 12 h vs Control 12 h	Ordinary two-way ANOVA (time × treatment); Šídák-adjusted prespecified comparisons	6.40554E-12
Figure 5	A	Viability	UVA 24 h vs Control 24 h	Ordinary two-way ANOVA (time × treatment); Šídák-adjusted prespecified comparisons	4.44089E-15
Figure 5	A	Viability	UVA 24 h vs UVA 4 h	Ordinary two-way ANOVA (time × treatment); Šídák-adjusted prespecified comparisons	5.67499E-08
Figure 5	A	Viability	UVA 24 h vs UVA 12 h	Ordinary two-way ANOVA (time × treatment); Šídák-adjusted prespecified comparisons	3.00365E-07
Figure 5	C	ROS	UVA 4 h vs Control 4 h	Ordinary two-way ANOVA (time × treatment); Šídák-adjusted prespecified comparisons	1.06581E-14
Figure 5	C	ROS	UVA 12 h vs Control 12 h	Ordinary two-way ANOVA (time × treatment); Šídák-adjusted prespecified comparisons	1.77636E-15
Figure 5	C	ROS	UVA 24 h vs Control 24 h	Ordinary two-way ANOVA (time × treatment); Šídák-adjusted prespecified comparisons	6.27336E-17
Figure 5	C	ROS	UVA 24 h vs UVA 4 h	Ordinary two-way ANOVA (time × treatment); Šídák-adjusted prespecified comparisons	6.59992E-07
Figure 5	C	ROS	UVA 24 h vs UVA 12 h	Ordinary two-way ANOVA (time × treatment); Šídák-adjusted prespecified comparisons	0.000206761
Figure 5	E	LPO	UVA 4 h vs Control 4 h	Ordinary two-way ANOVA (time × treatment); Šídák-adjusted prespecified comparisons	1.54707E-09
Figure 5	E	LPO	UVA 12 h vs Control 12 h	Ordinary two-way ANOVA (time × treatment); Šídák-adjusted prespecified comparisons	1.10383E-07
Figure 5	E	LPO	UVA 24 h vs Control 24 h	Ordinary two-way ANOVA (time × treatment); Šídák-adjusted prespecified comparisons	8.88178E-15
Figure 5	E	LPO	UVA 24 h vs UVA 4 h	Ordinary two-way ANOVA (time × treatment); Šídák-adjusted prespecified comparisons	1.12987E-09

Figure	Panel	Endpoint	Comparison	Statistical test / adjustment	Adjusted P
Figure 5	E	LPO	UVA 24 h vs UVA 12 h	Ordinary two-way ANOVA (time × treatment); Šídák-adjusted prespecified comparisons	3.87512E-11
Figure 5	G	GPX4	UVA 4 h vs Control 4 h	Ordinary two-way ANOVA (time × treatment); Šídák-adjusted prespecified comparisons	2.87455E-06
Figure 5	G	GPX4	UVA 12 h vs Control 12 h	Ordinary two-way ANOVA (time × treatment); Šídák-adjusted prespecified comparisons	0.012260099
Figure 5	G	GPX4	UVA 24 h vs Control 24 h	Ordinary two-way ANOVA (time × treatment); Šídák-adjusted prespecified comparisons	9.75598E-09
Figure 5	G	GPX4	UVA 24 h vs UVA 4 h	Ordinary two-way ANOVA (time × treatment); Šídák-adjusted prespecified comparisons	0.017390905
Figure 5	G	GPX4	UVA 24 h vs UVA 12 h	Ordinary two-way ANOVA (time × treatment); Šídák-adjusted prespecified comparisons	3.83212E-06
Figure 5	H	Fe^{2+}	UVA 4 h vs Control 4 h	Ordinary two-way ANOVA (time × treatment); Šídák-adjusted prespecified comparisons	5.55112E-16
Figure 5	H	Fe^{2+}	UVA 12 h vs Control 12 h	Ordinary two-way ANOVA (time × treatment); Šídák-adjusted prespecified comparisons	5.55112E-16
Figure 5	H	Fe^{2+}	UVA 24 h vs Control 24 h	Ordinary two-way ANOVA (time × treatment); Šídák-adjusted prespecified comparisons	5.55112E-16
Figure 7	A	Viability	UVA vs Control	Ordinary one-way ANOVA; Šídák-adjusted prespecified comparisons	1.73276E-09
Figure 7	A	Viability	UVA+SR8278 vs Control	Ordinary one-way ANOVA; Šídák-adjusted prespecified comparisons	1.51891E-07
Figure 7	A	Viability	UVA+SR8278 vs UVA	Ordinary one-way ANOVA; Šídák-adjusted prespecified comparisons	1.28763E-06
Figure 7	C	REV-ERBα	SR8278 vs Control	Ordinary one-way ANOVA; Šídák-adjusted prespecified comparisons	4.3764E-05
Figure 7	C	REV-ERBα	UVA+SR8278 vs Control	Ordinary one-way ANOVA; Šídák-adjusted prespecified comparisons	4.25382E-07
Figure 7	C	REV-ERBα	UVA+SR8278 vs UVA	Ordinary one-way ANOVA; Šídák-adjusted prespecified comparisons	1.93592E-06
Figure 7	C	GPX4	UVA vs Control	Ordinary one-way ANOVA; Šídák-adjusted prespecified comparisons	4.61457E-05
Figure 7	C	GPX4	UVA+SR8278 vs Control	Ordinary one-way ANOVA; Šídák-adjusted prespecified comparisons	0.0201647

Figure	Panel	Endpoint	Comparison	Statistical test / adjustment	Adjusted P
Figure 7	C	GPX4	UVA+SR8278 vs UVA	Ordinary one-way ANOVA; Šídák-adjusted prespecified comparisons	0.0015142
Figure 7	E	DCFH-DA	SR8278 vs Control	Ordinary one-way ANOVA; Šídák-adjusted prespecified comparisons	1.05379E-05
Figure 7	E	DCFH-DA	UVA vs Control	Ordinary one-way ANOVA; Šídák-adjusted prespecified comparisons	5.2799E-10
Figure 7	E	DCFH-DA	UVA+SR8278 vs Control	Ordinary one-way ANOVA; Šídák-adjusted prespecified comparisons	3.17888E-07
Figure 7	E	DCFH-DA	UVA+SR8278 vs UVA	Ordinary one-way ANOVA; Šídák-adjusted prespecified comparisons	5.69124E-08
Figure 7	G	Liperfluo	UVA vs Control	Ordinary one-way ANOVA; Šídák-adjusted prespecified comparisons	7.76046E-13
Figure 7	G	Liperfluo	UVA+SR8278 vs Control	Ordinary one-way ANOVA; Šídák-adjusted prespecified comparisons	7.67672E-08
Figure 7	G	Liperfluo	UVA+SR8278 vs UVA	Ordinary one-way ANOVA; Šídák-adjusted prespecified comparisons	6.64735E-12
Figure 7	H	Fe^{2+}	UVA vs Control	Ordinary one-way ANOVA; Šídák-adjusted prespecified comparisons	9.66533E-06
Figure 7	H	Fe^{2+}	UVA+SR8278 vs Control	Ordinary one-way ANOVA; Šídák-adjusted prespecified comparisons	1.68229E-05

Note: Adjusted P values were calculated using the statistical test and multiple-comparison procedure specified for each experiment.

The value of m denotes the number of prespecified comparisons included in the Šídák adjustment family.
